# The potential of FCRL genes as targets for cancer treatment: insights from bioinformatics and immunology

**DOI:** 10.18632/aging.204766

**Published:** 2023-06-02

**Authors:** Xiao Liang, Lei Du, Yuchao Fan

**Affiliations:** 1 Department of Anesthesiology, West China Hospital, Sichuan University, Chengdu, Sichuan Province, China; 2 Department of Anesthesiology, Sichuan Clinical Research Center for Cancer, Sichuan Cancer Hospital and Institute, Sichuan Cancer Center, Affiliated Cancer Hospital of University of Electronic Science and Technology of China, Chengdu, Sichuan Province, China

**Keywords:** Fc receptor-like, immunocytes, pan-cancer, immunotherapy, immune checkpoint, tumor microenvironment

## Abstract

Cancer is a prevalent and dangerous disease that requires a multifaceted approach to treatment. The FCRL family gene has been linked to immune function and tumor progression. Bioinformatics may help unravel their role in cancer treatment. We conducted a comprehensive analysis of the FCRL family genes in pan-cancer using publicly available databases and online tools. Specifically, we examined gene expression, prognostic significance, mutation profiles, drug resistance, as well as biological and immunomodulatory roles. Our data were sourced from The Cancer Genome Atlas, Genotype-Tissue Expression, cBioPortal, STRING, GSCALite, Cytoscape, and R software. The expression of FCRL genes varies significantly across different tumor types and normal tissues. While high expression of most FCRL genes is associated with a protective effect in many cancers, FCRLB appears to be a risk factor in several types of cancer. Alterations in FCRL family genes, particularly through amplification and mutation, are common in cancers. These genes are closely linked to classical cancer pathways such as apoptosis, epithelial-mesenchymal transition (EMT), estrogen receptor (ER) signaling, and DNA damage response. Enrichment analysis indicates that FCRL family genes are predominantly associated with immune cell activation and differentiation. Immunological assays demonstrate a strong positive correlation between FCRL family genes and tumor-infiltrating lymphocytes (TILs), immunostimulators, and immunoinhibitors. Furthermore, FCRL family genes can enhance the sensitivity of various anticancer drugs. The FCRL family genes are vital in cancer pathogenesis and progression. Targeting these genes in conjunction with immunotherapy could enhance cancer treatment efficacy. Further research is required to determine their potential as therapeutic targets.

## INTRODUCTION

Cancer is a complex and multifaceted disease that has been extensively studied by the scientific community due to its high prevalence and morbidity rates. It affects millions of people worldwide. In the United States alone, 1,958,310 new cases and 609,820 deaths are predicted for 2023 [1]. The incidence of cancer is influenced by numerous factors, such as genetics, lifestyle, environmental exposures, and aging. The peril of cancer lies in its capacity to rapidly disseminate and infiltrate neighboring tissues, resulting in metastasis and resistance to treatment. The treatment of cancer is a complex process that involves various modalities such as chemotherapy, radiation, immunotherapy, and surgery [2]. In recent years, the field of cancer research has made tremendous progress in developing new and innovative treatment options, such as targeted therapies and precision medicine. Exploring and developing new cancer treatments is of utmost importance, as it can significantly improve patient outcomes and quality of life, ultimately leading to a reduction in cancer-related morbidity and mortality [3].

Fc Receptor-like (FCRL) genes are a family of genes that play a role in the immune system by encoding proteins that function as receptors on the surface of immune cells [4]. These receptors are known to interact with antibodies and other molecules involved in the immune response [5]. Recent research has suggested that FCRL genes may play a role in the development and progression of various types of tumors. Multiple studies have identified a potential association between FCRL1-5 and B-cell malignancies. The immunomodulatory effects of FCRL1-5 highlight their potential as candidates for targeted therapy, diagnosis, and prognosis in relevant patients [6]. Moreover, FCRL6 has been shown to play a mechanistic role in immune checkpoint therapy-induced evasion in HLA-DR+ tumor samples from patients with recurrent melanoma, breast, and lung cancers after PD-1 blockade [7]. In addition, highly expressed FCRL genes have been linked to good overall survival (OS) in skin cutaneous melanoma (SKCM), indicating their potential as a biomarker for predicting prognosis in SKCM patients [8]. On the other hand, FCRLB has been significantly upregulated in colorectal cancer, suggesting its potential as a biomarker for this cancer type [9]. These findings demonstrate the FCRL gene family represents a promising therapeutic target in the treatment of tumors and the restoration of immune function in cancer patients. Further studies are required to fully understand the mechanism of action of FCRL genes and the precise role they play in shaping the immune microenvironment of tumors.

While the precise mechanisms responsible for the involvement of FCRL genes in tumorigenesis remain incompletely understood, bioinformatics techniques can offer valuable insights into their functional roles. By leveraging bioinformatics tools, researchers can gain a deeper understanding of the molecular mechanisms underlying the dysregulation of FCRLs in cancers, and how this phenomenon may contribute to the regulation of the tumor microenvironment. The use of bioinformatics in studying the role of FCRLs in tumor immunity has the potential to yield significant advances in cancer treatment and improve patient outcomes.

## RESULTS

### Expression of FCRL family mRNA in pan-cancer

**Figure 1:**
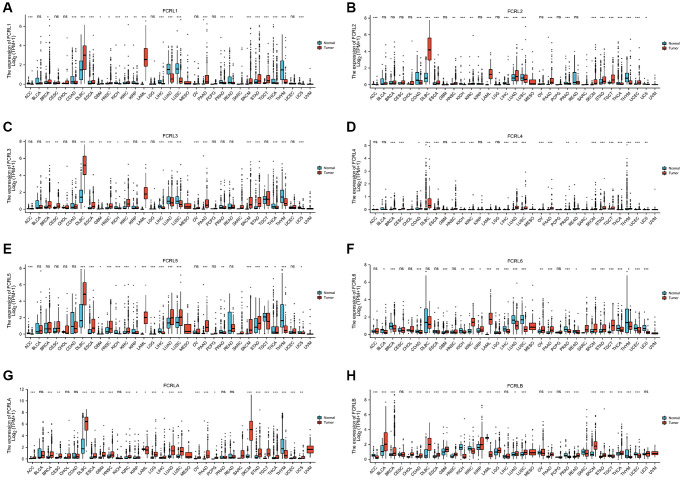
**mRNA expression profile of FCRL family gene in 33 cancers.** Expression of (**A**) FCRL1, (**B**) FCRL2, (**C**) FCRL3, (**D**) FCRL4, (**E**) FCRL5, (**F**) FCRL6, (**G**) FCRLA, (**H**) FCRLB mRNA in 33 cancers and normal tissues in unpaired sample analysis. ^∗^*p* < 0.05, ^∗∗^*p* < 0.01, ^∗∗∗^*p* < 0.001.

A total of 15776 individuals from The Cancer Genome Atlas (TCGA) and Genotype-Tissue Expression (GTEx) were included in the unpaired sample comparison. FCRL1, FCRL2, and FCRL4 are low in both most tumors and normal tissues. As Figure 1A showed, FCRL1 was expressed significantly lower in ACC, COAD, KICH, KIRC, KIRP, LIHC, LUAD, LUSC, PRAD, READ, THCA, THYM, and UCS tumor tissue than in normal tissue and significantly higher in BRCA, DLBC, ESCA, GBM, HNSC, LAML, PAAD, SKCM, STAD, and TGCT than in normal tissue. FCRL2 expression was significantly higher in LUAD, DLBC, ESCA, KIRC, HNSC, LAML, PAAD, SKCM, STAD, TGCT, and UCEC but significantly lower in ACC, COAD, KICH, KIRP, LIHC, LUSC, PRAD, THCA, THYM, UCS compared to normal tissue (Figure 1B). FCRL3 expression was significantly higher in BRCA, CESC, DLBC, ESCA, GBM, HNSC, KIRC, LAML, OV, PAAD, SKCM, and STAD but significantly lower in KICH, LIHC, LUAD, LUSC, THCA, THYM, UCS compared to normal tissue (Figure 1C). FCRL4 expression was significantly higher in BRCA, CESC, COAD, DLBC, ESCA, LUSC, HNSC, LUAD, PAAD, READ, STAD, TGCT, THCA, THYM, UCEC but significantly lower in KICH, KIRC, LAML, LIHC, OV, PRAD, SKCM, UCS compared to normal tissue (Figure 1D). FCRL5 expression was significantly higher in DLBC, ESCA, HNSC, KIRC, LAML, LUAD, LUSC, PAAD, READ, SKCM, and STAD but significantly lower in ACC, GBM, KICH, KIRP, LGG, LIHC, PRAD, TGCT, THCA, THYM, UCS compared to normal tissue (Figure 1E). FCRL6 expression was significantly higher in GBM, KIRC, KIRP, LAML, OV, PAAD, SKCM, STAD, and TGCT but significantly lower in BRCA, BLCA, COAD, KICH, LGG, LIHC, LUAD, LUSC, PRAD, READ, THCA, THYM, UCS, UCEC compared to normal tissue (Figure 1F). FCRLA expression was significantly higher in ACC, CESC, BRCA, DLBC, ESCA, GBM, HNSC, KIRC, KIRP, LGG, LIHC, LUAD, LUSC, OV, PAAD, PCPG, SKCM, STAD, TGCT, THCA, UCEC, UCS but significantly lower in COAD, KICH, LAML, THYM compared to normal tissue (Figure 1G). FCRLB expression was significantly higher in BLCA, BRCA, CHOL, DLBC, ESCA, GBM, HNSC, KIRP, LGG, LUAD, LUSC, PAAD, READ, SKCM, STAD, THCA but significantly lower in ACC, COAD, KICH, KIRC, LAML, LIHC, PRAD, TGCT, UCEC, THYM compared to normal tissue (Figure 1H). The expression of FCRL family genes in MESO cannot be compared with normal tissues due to the lack of data in the corresponding normal tissues.

### Prognostic value of the FCRL family gene in pan-cancer

**Figure 2:**
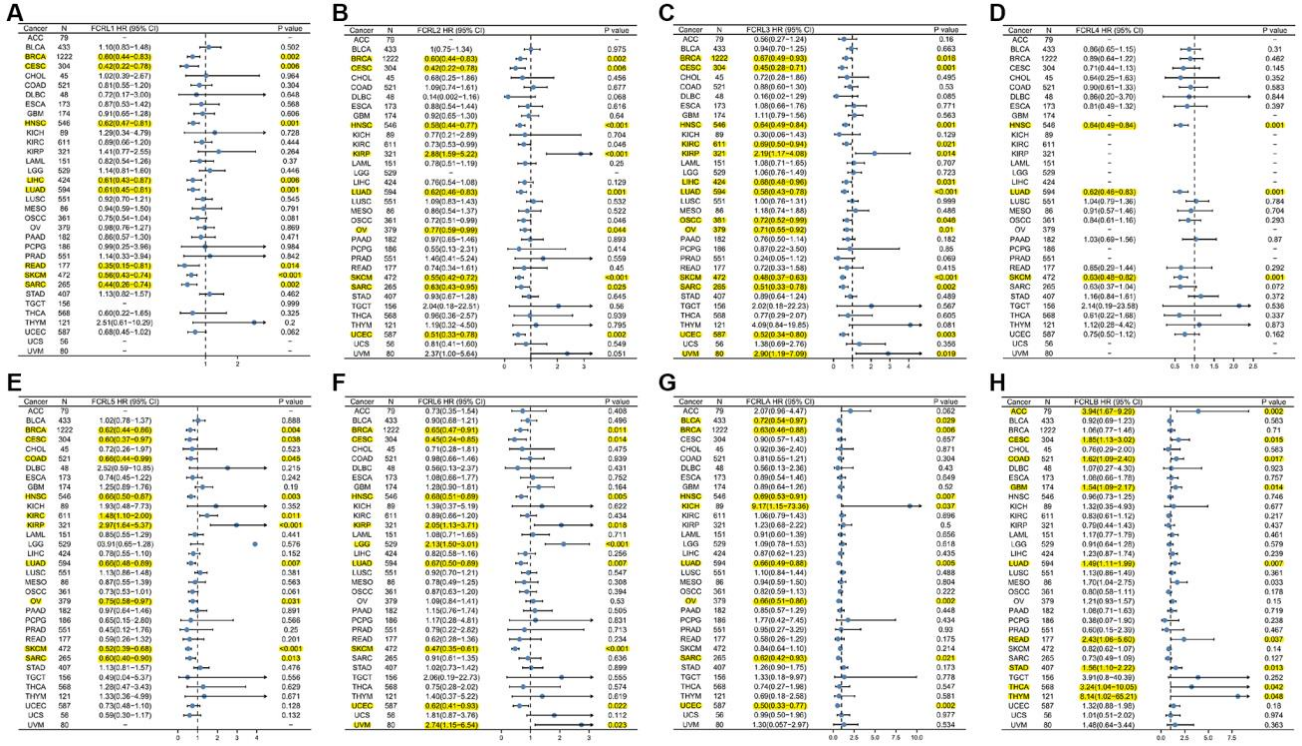
**Prognostic value of the FCRL family gene in pan-cancer.** Forest plot of (**A**) FCRL1, (**B**) FCRL2, (**C**) FCRL3, (**D**) FCRL4, (**E**) FCRL5, (**F**) FCRL6, (**G**) FCRLA, (**H**) FCRLB OS in 34 cancers. The FCRL gene has been identified as a potential protective or risk factor in the development of this particular cancer, as indicated by its highlighted representation in yellow.

We used Kaplan–Meier (K-M) analysis to evaluate the prognostic value of the FCRL family gene in pan-cancer. High FCRL1 showed statistically superior OS in BRCA, CESC, HNSC, LIHC, LUAD, READ, SKCM, and SARC (Figure 2A). High FCRL2 groups showed a statistically superior OS to low FCRL2 group in BRCA, CESC, HNSC, KIRC, LUAD, OSCC, OV, READ, SKCM, SARC, UCEC, and a statistically worse OS than low FCRL2 group in KIRP (Figure 2B). High FCRL3 was discovered to be a protective variable in BRCA, CESC, HNSC, KIRC, LIHC, LUAD, OSCC, OV, SKCM, SARC, and UCEC and a risk variable in KIRP, UVM (Figure 2C). High FCRL4 has better OS than low FCRL4 in HNSC, LUAD, and SKCM (Figure 2D). As shown in Figure 2E, high FCRL5 is a protective factor for BRCA, CESC, HNSC, LUAD, OV, SKCM, SARC, and a risk factor for KIRC and KIRP. High FCRL6 is a protective factor for BRCA, CESC, HNSC, LUAD, and SKCM and a risk factor for LGG, KIRP, and UVM (Figure 2F). High FCRLA groups showed a statistically superior OS than the low FCRLA group in BLCA, BRCA, HNSC, LUAD, OV, SARC, and UCEC and a statistically worse OS than low FCRLA group in KICH and LGG (Figure 2G). Unlike other FCRL family genes where high expression is a protective factor for a variety of cancers, high FCRLB is a risk factor for ACC, CESC, COAD, GBM, LUAD, MESO, READ, STAD, THCA, and THYM (Figure 2H).

### Genetic alteration of FCRL family gene

**Figure 3:**
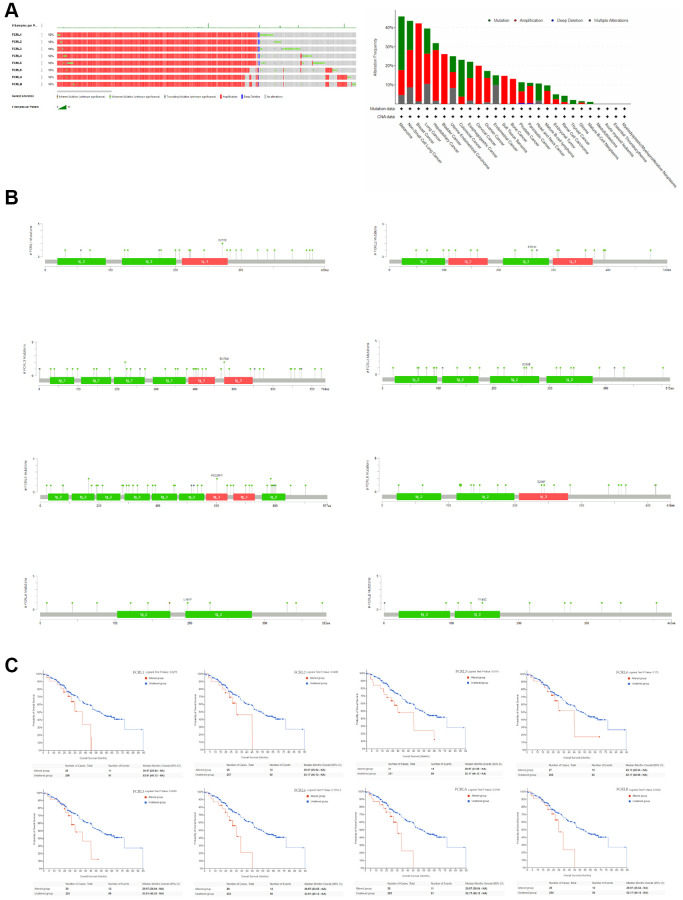
**Genetic alteration of FCRL family gene.** (**A**) Bar chart of FCRL family gene mutation in pan-cancer based on ICGC/TCGA database, and the alteration frequency with different types of FCRL family gene mutations in pan-cancer. (**B**) Mutation diagram of FCRL family gene across protein domains. (**C**) Survival analysis of OS based on altered and unaltered FCRL family gene.

The genetic alterations affecting the expression of FCRL family genes in pan-cancer were examined using the cBioPortal web tool. The analysis comprised 2922 samples from the ICGC/TCGA databases. The most frequently occurring genetic modifications were amplification and mutation. The frequency of genetic variants was 13%, 13%, 14%, 13%, 13%, 13%, 13%, and 13% in FCRL1-6, A, and B, respectively. The frequency of alterations in FCRL family genes exceeded 20% in melanoma, non-small cell lung cancer, breast cancer, lung cancer, hepatobiliary cancer, bladder cancer, endometrial cancer, colorectal cancer, and esophageal cancer, and the main types of alterations were mutations and amplifications (Figure 3A). The specific alteration sites between amino acids for each FCRL family gene were illustrated in Figure 3B. The unaltered group of FCRL1-3, 5, 6, and A had better OS compared to the altered group (Figure 3C).

### The copy number variation (CNV), single nucleotide variation (SNV), and methylation of FCRL family genes in different cancer type

**Figure 4:**
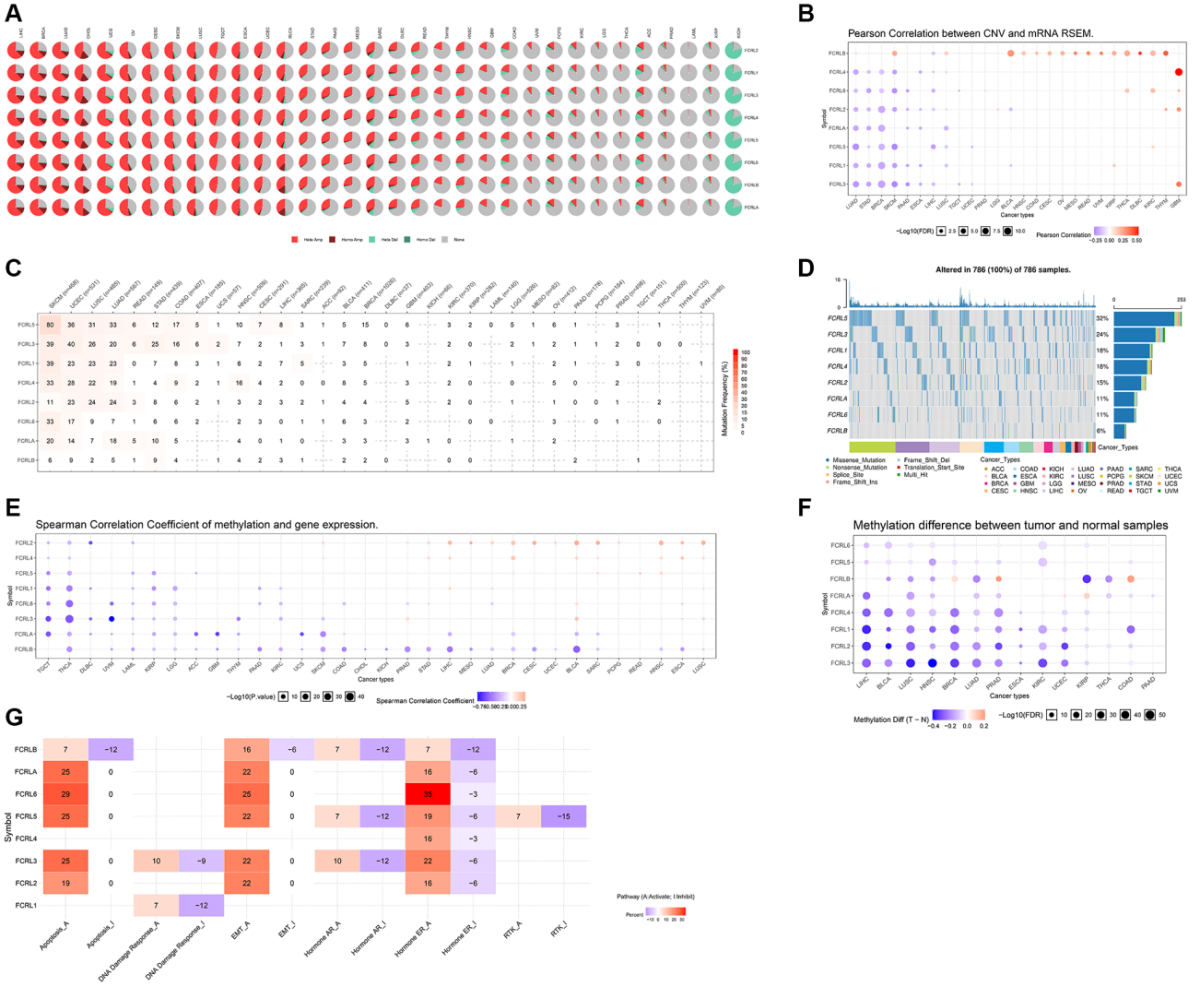
**CNV, SNV and Methylation of FCRL family genes in pan-cancer.** (**A**) CNV profiles of 33 cancer types’ FCRL family gene expression. Homo Amp stands for homozygous amplification, while Hete Amp and Hete Del stand for heterozygous amplification and deletion, respectively. (**B**) Correlation between CNV and the mRNA expression of genes from the FCRL family in 26 different cancer types. The darker the colour, the higher the correlation. Blue bubbles reflect a negative correlation, whereas red bubbles show a positive correlation. Each bubble's size indicates statistical significance. (**C**) SNV percentage profile of 32 cancer types’ associated FCRL family genes. (**D**) SNV frequency of genes from the FCRL family in all cancers. Patients are represented by the grey vertical bars in the graph. The number of variations per sample or in each gene is shown in the diagrams in the top and side columns. (**E**) Correlation between the expression levels of the genes in the FCRL family and their methylation status. Positive correlation is represented by red, while negative correlation is represented by blue. (**F**) Various FCRL family gene methylation patterns in 14 malignancies and healthy tissues. The darker the dots, the larger the changes in methylation up- and down-regulation in tumours are represented by blue and red dots. Statistics are indicated by the size of the dots. (**G**) Overall FCRL family gene pathway activity in 33 different cancer types. I stands for Inhibit, and A is for Activate.

We used GSCALite, a comprehensive analysis platform, to analyze the CNV, SNV, Methylation, and Pathway Activity of FCRL family genes in pan-cancer. The pie chart showed that the FCRL family genes were amplified heterozygously for CNV types in most cancers, especially LIHC, BRCA, LUAD, CHOL, UCS, OV, CESC, SKCM, LUSC, TGCT, ESCA, UCEC, and BLCA. Homozygous amplification of FCRL family genes was more common in LIHC, BRCA, LUAD, and CHOL. In specific, heterozygous deletions of FCRL family genes were dominant in KICH (Figure 4A). Furthermore, CNV and FCRL family gene expression correlations revealed that FCRLB expression was positively correlated with CNV in a variety of tumors. In contrast, expression of FCRL1-5 and A was found to be negatively associated with CNV in a variety of cancer types including LUAD, STAD, BRCA, SKCM, PAAD, ESCA, LIHC, and LUSC (Figure 4B).

SNV analysis revealed a high prevalence of mutations in FCRL1-5 and A in SKCM, UCEC, LUSC, and LUAD. FCRLB mutations were uncommon in almost all tumors. Missense mutations were the most common type of mutation in FCRL family genes in pan-cancer (Figure 4C, 4D).

Correlation analysis of methylation levels and mRNA expression levels revealed that in most tumors, mRNA expression of FCRLA and B was negatively correlated with methylation levels. The mRNA expression of FCRL family genes was mainly negatively correlated with their DNA methylation levels in TGCT, THCA, DLBC, UVM, LAML KIRP, and LGG (Figure 4E). The GSCALite platform was used to analyze the DNA methylation differences of FCRL family genes between tumors and normal tissues in various cancers, and it was discovered that FCRL1-4 and A methylation were significantly down-regulated in LIHC, BLCA, LUSC, HNSC, BRCA, LUAD, PRAD, MESC, KIRC, and UCEC (Figure 4F).

### The pathway activity and inhibition of FCRL family genes in pan-cancer

The linked pathway network revealed that FCRL family genes were involved in nine well-known cancer-related signaling pathways. FCRL2, 3, 5, 6, A, and B are mostly involved in apoptosis and epithelial–mesenchymal transition (EMT) activation. FCRL2-6, A, and B are associated with both activation and inhibition of the hormone estrogen receptor (ER). FCRL1 is only associated with activation and inhibition of the DNA damage response (Figure 4G).

### The PPI network of FCRL family and related hub genes

The investigation utilized the STRING database as a primary source to acquire relevant proteins belonging to the FCRL family and to subsequently establish a Protein-Protein Interaction (PPI) network via Cytoscape. The analysis of each PPI network was performed to identify the hub genes within the network for each gene of the FCRL family, utilizing the MCC algorithm available through the CytoHubba plugin integrated within the Cytoscape. Top 10 hub genes in FCRL1’s PPI network are FCRL1, FCGR2B, PTPN6, PTPN11, LAIR1, FCGR3A, FCGR1A, OBBP2, ENSP00000470259, and PIGR. For FCRL2 are FCRL2, FCGR2B, CD79A, CD79B, CD72, PTPN6, MS4A1, FCGR2A, TCL1A, and CD1E. For FCRL3 are FCRL3, CTLA4, PTPN22, HLA-DRB1, IL2RA, CD40, STAT4, TRAF1, ZAP70, and SYK. For FCRL4 are FCRL4, IGHV4-38-2, CD19, CR2, MME, CD27, CD38, C1GALT1, C1GALT1C1, and B4GALT1. For FCRL5 are FCRL5, CD19, CD27, CD38, SDC1, PTPRC, CR2, SLAMF7, TNFRSF17, and SYK. For FCRL6 are FCRL6, GZMB, NKG7, GZMH, CTSW, KLRF1, CD244, ZNF683, LAG3, and PTPN11. For FCRLA are FCRLA, CD19, CD79B, CD79A, MS4A1, CR2, CD22, TNFRSF13C, POU2AF1, and CD27. For FCRLB are FCRLB, KIRREL, GP6, FCGRT, CD5L, CD1E, GFRAL, CA3, VN1R1, and ZNF646 (Supplementary Figure 1A, 1B).

### Gene ontology (GO) and Kyoto Encyclopedia of Genes and Genomes (KEGG) functional enrichment of FCRL family genes

**Figure 5:**
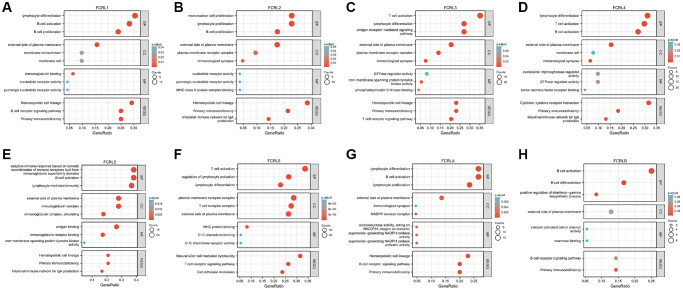
**GO and KEGG functional enrichment of FCRL family genes.** GO and KEGG functional enrichment of (**A**) FCRL1, (**B**) FCRL2, (**C**) FCRL3, (**D**) FCRL4, (**E**) FCRL5, (**F**) FCRL6, (**G**) FCRLA, (**H**) FCRLB. Abbreviations: BP: biological process; MF: molecular function; CC: cellular component.

The top 100 mRNA that interact closely with each FCRL family gene were used for GO/KEGG enrichment analysis. Three kinds of RNA function were identified: the biological process (BP), molecular function (MF), and cellular component (CC). The common BP terms were B cell proliferation, lymphocyte proliferation, lymphocyte differentiation, T cell activation, and B cell activation; the T cell activation was also shown in BP of FCRL3, 4, and 6. The common CC terms were external side of plasma membrane, membrane raft, and immunological synapse; the MF terms were mostly associated with nucleotide receptor activation for FCRL1 and 2 and GTPase regulator activity for FCRL3, 4. Antigen and immunoglobulin receptor binding were MF terms associated with FCRL5. MFs that are closely related to FCRL6 are MHC protein binding, C-C chemokine binding, and C-C chemokine receptor activity. MF of FCRLA is associated with superoxide-generating NADPH oxidase activity. KEGG pathways associated with FCRL family genes included Hematopoietic cell lineage, primary immunodeficiency, intestinal immune network for IgA production, and T and B cell receptor signaling pathway. Notably, KEGG in FCRL6 is also associated with natural killer cell-mediated cytotoxicity (Figure 5A–5H).

### Gene set enrichment analysis (GSEA)

**Figure 6:**
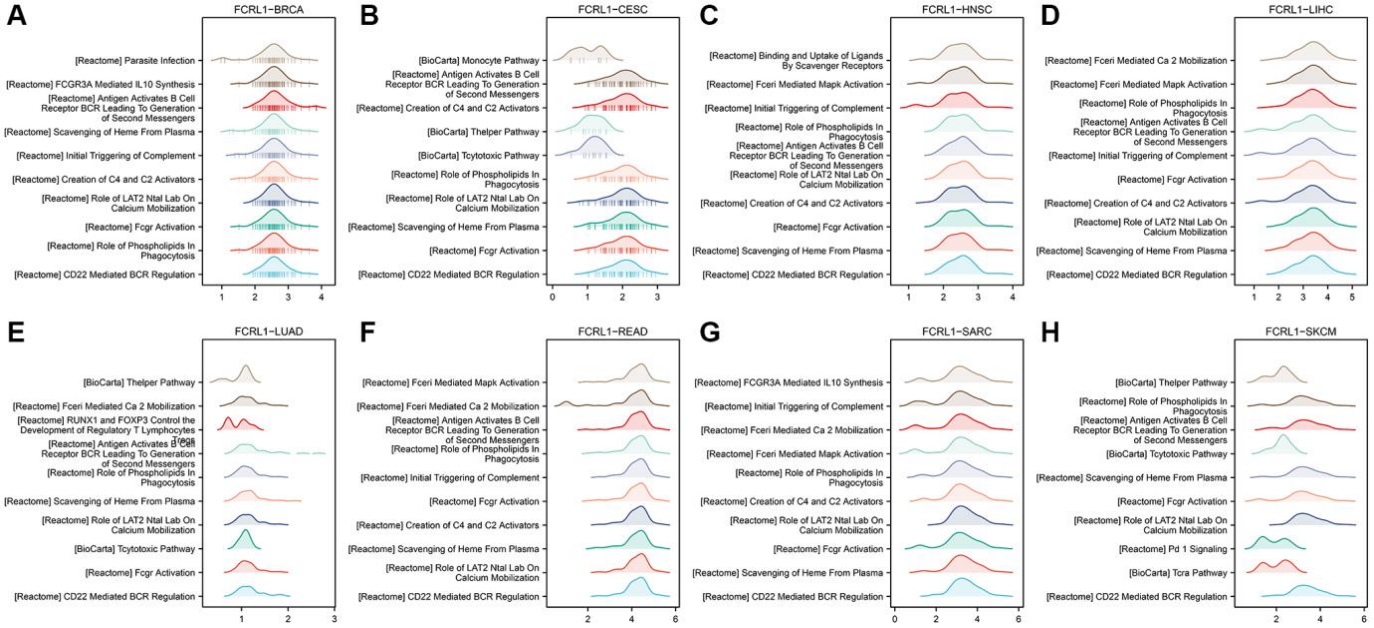
**GESA for DEGs of high- and low-FCRL1 group in different cancer types.** (**A**) BRCA, (**B**) CESC, (**C**) HNSC, (**D**) LIHC, (**E**) LUAD, (**F**) READ, (**G**) SARC, (**H**) SKCM.

We performed GSEA for each FCRL family gene in cancers in which expression of FCRL family genes significantly affected OS. The top 10 enriched pathways of GSEA for FCRL1 in BRCA, CESC, HNSC, LIHC, LUAD, READ, SKCM, and SARC were shown in Figure 6A–6H. The enriched terms that CD22 mediated B cell receptor (BCR) regulation, Fcγ receptors activation, role of LAT2, NTAL, and LAB on calcium mobilization, scavenging of heme from plasma, creation of C4 and C2 activators, antigen activates BCR leading to generation of second messengers, role of phospholipid in phagocytosis had shown in 5 cancers and initial triggering of complement had shown in 4 cancers. The top 10 enriched terms of other FCRL family genes were shown in Supplementary Table 1.

### Relationship between FCRL family gene and tumor immune microenvironment in pan-cancer

To examine the correlation between FCRL family genes and immune infiltration and regulation, heatmaps were constructed to illustrate the expression of FCRL genes alongside markers of Tumour-infiltrating lymphocytes (TILs), immunostimulators, and immunoinhibitors. Our findings revealed that, in the majority of cancer types, all FCRL family genes, with the exception of FCRLB, exhibited a positive correlation with TILs, immunostimulators, and immunoinhibitors.

**Figure 7:**
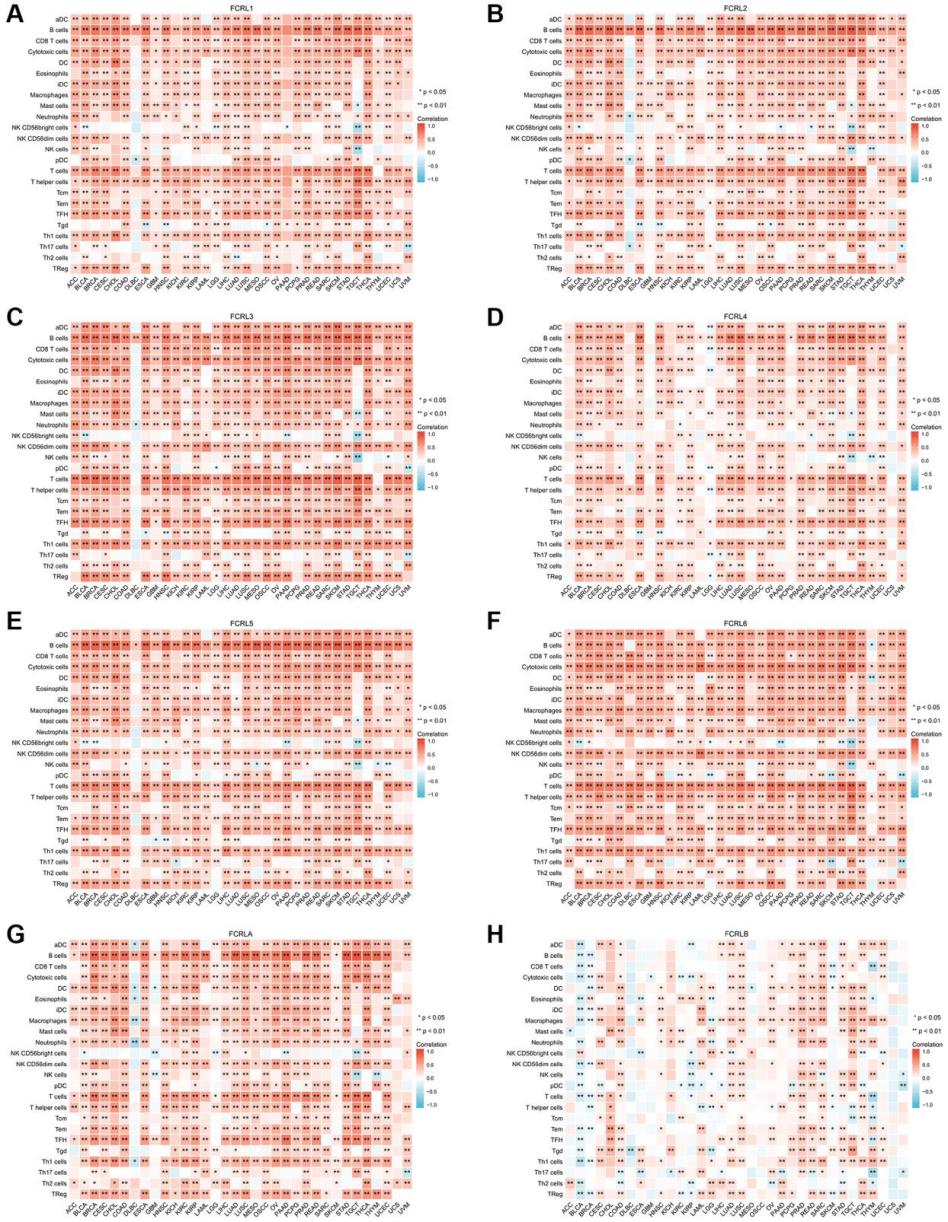
**Correlation of FCRL family genes with markers of TILs in pan-cancer.** Correlations between (**A**) FCRL1, (**B**) FCRL2, (**C**) FCRL3, (**D**) FCRL4, (**E**) FCRL5, (**F**) FCRL6, (**G**) FCRLA, (**H**) FCRLB expression and markers of TILs. ^∗^*p* < 0.05, ^∗∗^*p* < 0.01.

The expression of FCRL1-6 and A exhibited strong positive correlations with most TILs in various tumors, with the exception of the marker of natural killer CD56bright cells. This association was particularly prominent in the markers of B and T cells. However, in diffuse large B-cell lymphoma (DLBC), FCRL1-5 and A gene expression did not exhibit significant correlations with markers of other TILs, except for B cells, which demonstrated a strong positive correlation. Conversely, FCRL6 expression showed no significant correlation with B cells in DLBC but instead exhibited a strong positive correlation with several other markers of TILs (Figure 7A–7H).

**Figure 8:**
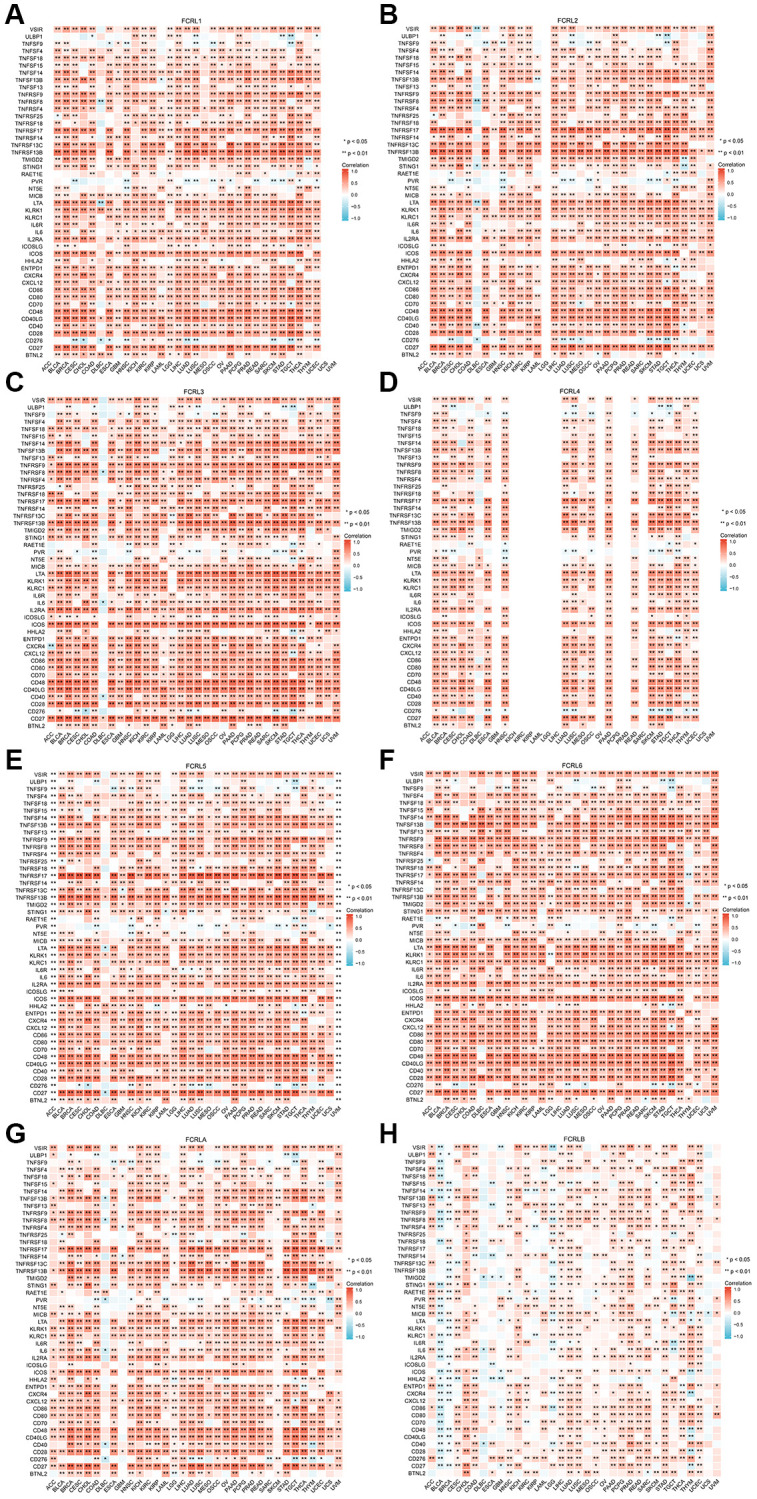
**Correlation of FCRL family genes with markers of immunostimulators in pan-cancer.** Correlations between (**A**) FCRL1, (**B**) FCRL2, (**C**) FCRL3, (**D**) FCRL4, (**E**) FCRL5, (**F**) FCRL6, (**G**) FCRLA, (**H**) FCRLB expression and markers of immunostimulators. ^∗^*p* < 0.05, ^∗∗^*p* < 0.01.

**Figure 9:**
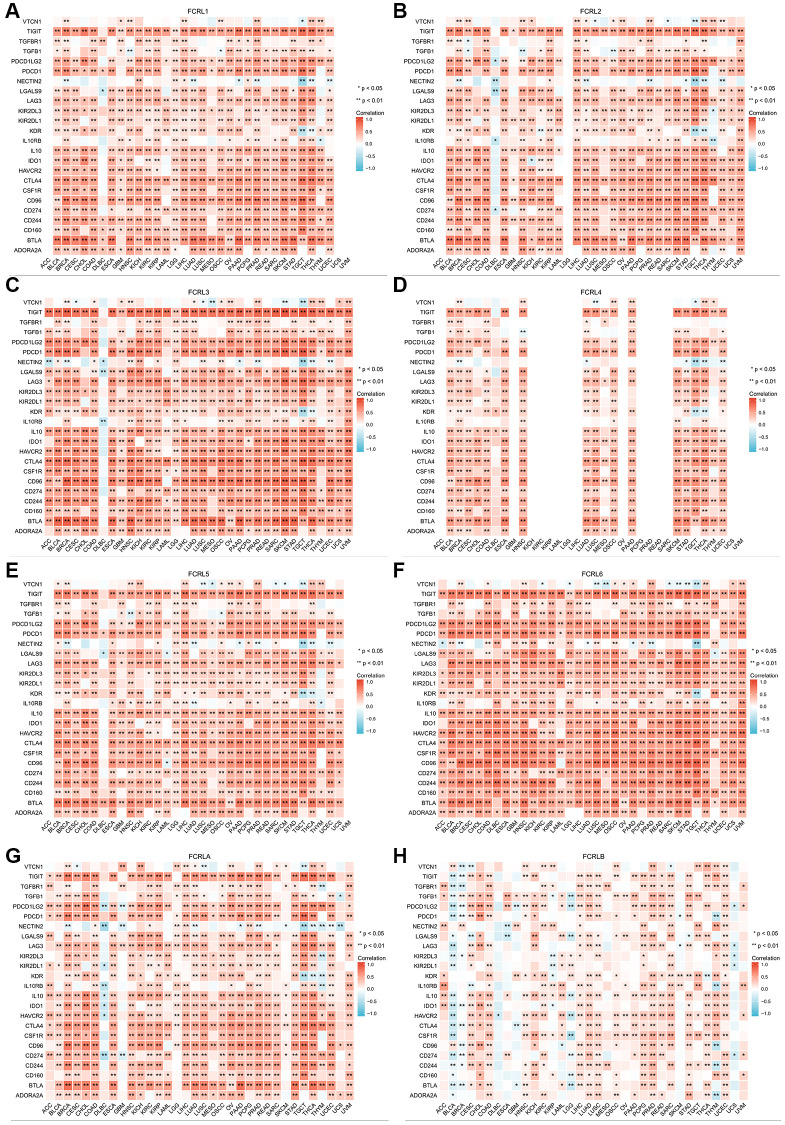
**Correlation of FCRL family genes with markers of immunoinhibitors in pan-cancer.** Correlations between (**A**) FCRL1, (**B**) FCRL2, (**C**) FCRL3, (**D**) FCRL4, (**E**) FCRL5, (**F**) FCRL6, (**G**) FCRLA, (**H**) FCRLB expression and markers of immunoinhibitors. ^∗^*p* < 0.05, ^∗∗^*p* < 0.01.

At the level of immunostimulators, the expression of FCRL1-6 and A demonstrated strong positive correlations with most markers of immunostimulators, with the exception of ULBP1, TNFSF9, RAET1E, PVR, ICOSLG, and CD276 (Figure 8A–8H). Similarly, the expression of FCRL1-6 and A in most tumors demonstrated strong positive correlations with most markers of immunoinhibitors, except for VTCN1, TGFBR1, TGFB1, NECTIN2, and IL10RB (Figure 9A–9H). Correlations between FCRL4 and markers of immunostimulators and immunoinhibitors for a number of tumours including GBM, KICH, KIRC, KIRP, LAML, LGG, LIHC, OV, PCPG, PRAD, READ, SARC, UCS, and UVM could not be analysed due to missing data in the database.

In DLBC, FCRL6 expression exhibited strong positive correlations with most markers of immunostimulators and immunoinhibitors, while other FCRL family genes did not demonstrate significant correlations with most markers of immunostimulators and immunoinhibitors. Specifically, FCRL1-3, 5, and 6 expressions exhibited negative correlations with certain markers of immunostimulators and immunoinhibitors (Figures 8, 9).

The expression of the FCRLB in various cancers has been found to be significantly associated with the markers related to TILs, immunostimulators, and immunoinhibitors. Specifically, in several cancers including bladder urothelial carcinoma (BLCA), breast invasive carcinoma (BRCA), kidney renal papillary cell carcinoma (KIRP), lower grade glioma (LGG), and uterine corpus endometrial carcinoma (UCS), the expression of FCRLB was negatively correlated with the certain TILs, immunostimulators, and immunoinhibitors markers. In contrast, in other cancers, FCRLB showed a comparatively weaker positive correlation with these markers as compared to other members of the FCRL gene family (Figures 7–9).

**Figure 10:**
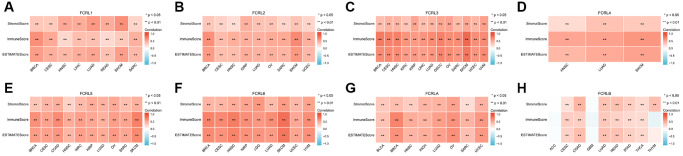
**Correlation between FCRL family gene expression and tumor purity in cancers impacting OS.** Correlations between (**A**) FCRL1, (**B**) FCRL2, (**C**) FCRL3, (**D**) FCRL4, (**E**) FCRL5, (**F**) FCRL6, (**G**) FCRLA, (**H**) FCRLB expression and Stromalscore, Immune score, and ESTIMATE score. ^∗^*p* < 0.05, ^∗∗^*p* < 0.01.

In our study, we analyzed the correlation between the expression of FCRL family genes and Stromalscore, Immunescore, and ESTIMATEscore in multiple types of cancer. The expression of FCRL family genes exerts a notable impact on the OS of these tumors. Our results indicated that FCRL family genes, with the exception of FCRLB, exhibited a significant and positive correlation with the aforementioned scores in the examined tumors (Figure 10A–10G). However, in Adrenocortical carcinoma (ACC) and Glioblastoma multiforme (GBM), the correlations between FCRLB gene expression and Stromalscore, Immunescore, and ESTIMATE score were found to be insignificant. Similarly, in Thymoma (THYM), the correlations between FCRLB gene expression and Immunescore and ESTIMATE score were also insignificant (Figure 10H).

### Drug sensitivity of FCRL family genes in pan-cancer

In this study, we investigated the relationship between FCRL family gene expression and the drug IC50 using the GSCALite online platform. IC50 data from two drug databases, Genomics of Drug Sensitivity in Cancer (GDSC) and Cancer Therapeutics Response Portal (CTRP), were utilized in the analysis. Spearman correlation analysis was performed to examine the expression of each FCRL family gene in pan-cancer and its association with different small molecule/drug sensitivity (IC50). The drugs obtained were ranked based on their relevance to the FCRL family using Spearman correlation analysis of the database.

**Figure 11:**
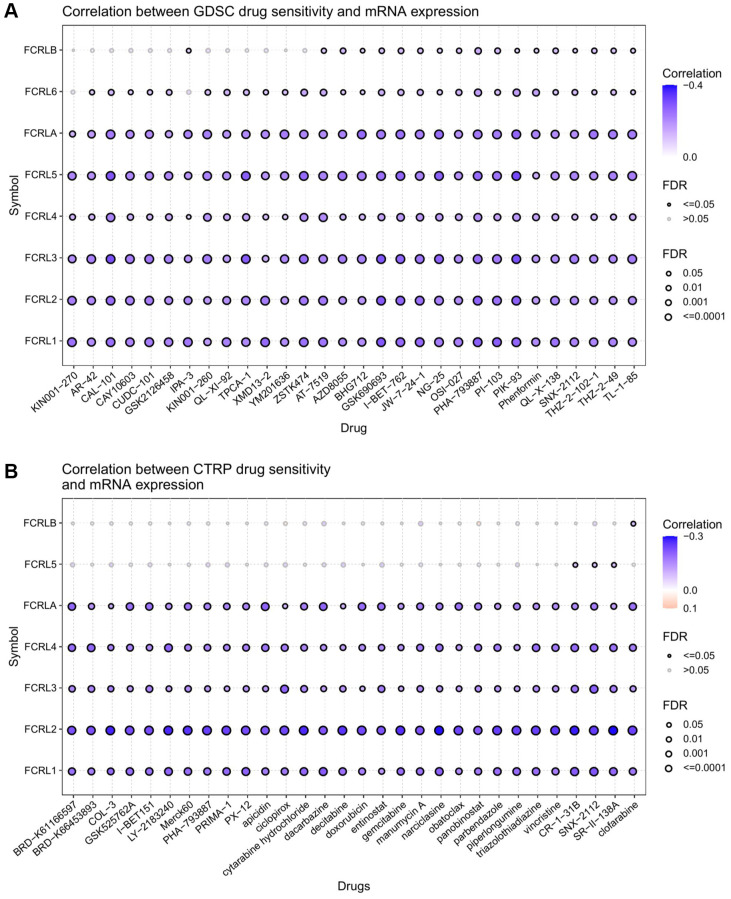
**Drug sensitivity analysis of FCRL family gene.** The correlation between FCRL family gene expression and drug sensitivity in (**A**) GDSC and (**B**) CTRP database. The correlation between gene expression and drug sensitivity was assessed using Pearson's correlation coefficient. Negative correlations were denoted by blue bubbles, while positive correlations were represented by red bubbles. The intensity of the color reflected the strength of the correlation, with darker hues indicating higher levels of correlation. The size of the bubbles was positively associated with the FDR significance, and bubbles outlined in black corresponded to an FDR < 0.05. Our analysis was limited to the top 30 ranked drugs.

The results obtained from the GDSC database demonstrated a negative correlation between FCRL family genes and various drugs in pan-cancer. Notably, FCRL4, 6, and B were found to be less negatively associated with drugs compared to other FCRL family genes (Figure 11A). On the other hand, the analysis of the CTRP database revealed a significant negative correlation between FCRL1-4 and A and all drugs. Additionally, FCRL5 was also negatively correlated with most drugs, albeit to a lesser extent, and exhibited a positive correlation with ciclopirox and Panobinostat (Figure 11B).

## DISCUSSION

The FCRL family gene is a group of genes that encode cell surface receptors involved in regulating immune cell activation and function [5]. The FCRL genes are primarily expressed in B cells, but some are also expressed in other immune cells [6, 10, 11]. FCRL receptors can interact with immunoglobulin (Ig) molecules and modulate signaling pathways downstream of the B cell receptor [12]. Several studies have investigated the role of FCRL genes in cancer. Aberrant expression of FCRL genes has been observed in various types of cancer, including leukemia, lymphoma, and solid tumors [7, 9, 13]. Some studies have suggested that FCRL may contribute to cancer cell proliferation and survival by regulating intracellular signaling pathways or modulating the immune response in the tumor microenvironment [8, 9, 14]. However, the exact role of FCRL genes in cancer remains unclear and requires further investigation. Here, we present the initial all-encompassing and thorough analysis of FCRL family genes in pan-cancer.

In this study, we conducted a comprehensive analysis of the expression, mutations, and functional roles of FCRL family genes in pan-cancer. Firstly, we assessed the expression of each member of the FCRL gene family across different cancer types and evaluated their impact on overall survival. We also analyzed the genetic alterations of FCRL genes, including copy number variations, single nucleotide variants, and methylation changes. To understand the functional implications of FCRL genes, we performed functional enrichment analyses, including GO and KEGG pathway analyses, as well as GSEA to identify cancer-related pathways modulated by FCRL family genes. Furthermore, we investigated the relationship between FCRL family gene expression and various immune-related factors, such as TILs, immunostimulators, and immunoinhibitors, as well as their association with the tumor microenvironment. Finally, we examined the association between FCRL family genes and drug sensitivity to identify potential therapeutic targets for cancer treatment.

We assessed the expression of FCRL family genes across various cancers. The findings revealed distinct expression patterns of FCRL genes in tumor versus normal tissues. The observed marked differences in expression suggest that FCRL genes may play a role in tumorigenesis and regulation in diverse cancers. Notably, within a particular tumor, elevated expression of certain gene family members was observed, while reduced expression of others was seen in another tumor. For instance, all FCRL family genes were upregulated in ESCA, whereas all genes except FCRLA were downregulated in KIRC. Such differential expression may be attributed to the varying distribution of immune cells in different tumors [15–17]. The composition and distribution of immune cells in the tumor microenvironment are known to significantly influence tumor progression, growth, and response to treatment [18].

The FCRL family exhibits a distinctive tissue distribution pattern [19], with FCRL1-5 and FCRLA/B being predominantly expressed by B cells. FCRL1 is expressed on all B cells, while FCRL2-5 are expressed on specific B cell subsets, and FCRLA/B are expressed by germinal center B cell subsets. FCRL6 is mainly expressed by T cells and NK cells [4], and FCRL3 is also expressed on circulating NK cells [20] and in regulatory T cell subsets [10]. These findings suggest that FCRL family genes may have varying roles in tumor immunity regulation through the distribution of immune cells in specific tumors. Notably, FCRL expression is limited in many organs, including both tumors and normal tissues. However, it is highly expressed in lymphatic system tumors such as DLBC, indicating that its expression is linked to the abundance of immune cells in the corresponding organ or tissue. This low expression in solid organs or tissues may hinder effective experiments to investigate the mechanisms of FCRL family genes involved in tumorigenesis and regulation in these sites. Consequently, current studies primarily focus on lymphatic system cancers, with fewer studies investigating cancers of other solid organs or tissues. To further explore the regulatory role of FCRL family genes in different types of tumors, particularly in solid organs or tissues where FCRL expression is low, new technical tools may be required.

The FCRL gene family has been shown to impact patient survival in a range of cancers, with FCRL1-6 and A acting as protective factors in many tumors. Upregulation of multiple FCRL family genes has been linked to improved prognosis in several tumor types, including BRCA, CESC, HNSC, LUAD, SKCM, and SARC. Conversely, certain tumors, such as KIRP and UVM, have been associated with an increased risk when FCRL family genes are upregulated. These findings suggest that FCRL family genes may play a similar or opposing role in regulating tumor progression, potentially through common or opposing mechanisms of invasion, metastasis, and immunosuppression. Notably, there is a high frequency of mutations in FCRL family genes in various cancers, particularly those in which FCRL family genes significantly affect overall survival, such as BRCA, LUAD, and CESC. Furthermore, the mutation of FCRL family genes also has an impact on the overall survival of pan-cancer, further supporting the key role of FCRL family genes in regulating tumor progression.

FCRLB is primarily expressed intracellularly and lacks any transmembrane region [21]. However, the investigation of FCRLB has been challenging due to its low level of transcripts that cannot be detected by Northern blot or even RT-PCR [22]. FCRLB exhibits a mutually exclusive expression pattern with FCRLA in B cells [23]. This may partially account for our findings that FCRLA serves as a protective factor in LUAD prognosis, while FCRLB poses a risk. Chikaev et al. have demonstrated significant expression of the FCRLB gene in transformed B-cell lines, whereas it is either absent or weakly expressed in normal B cells [24]. In addition, the expression of FCRLB is higher in malignant and metastatic melanomas compared to melanocytic nevi. Wilson et al. discovered that FCRLB was expressed in non-proliferating cells within the germinal center and postulated that it may deactivate these B cells, which could generate autoantibodies against either benign, non-self-antigens or antibodies unable to compete with neighboring cells for antigen binding [22]. Given the current dearth of studies on FCRLB, further research should elucidate its role in tumor immunity.

Our results indicate that FCRL family genes are associated with the activation or repression of multiple classical cancer-related pathways. Specifically, FCRL2, 3, 5, 6, A, and B are primarily involved in apoptosis and EMT activation. Apoptosis is critical for maintaining tissue homeostasis and preventing cancer development, and tumors can evade apoptosis through various mechanisms that allow their growth and survival [25]. Therapies that induce apoptosis in cancer cells are therefore an important strategy for cancer treatment [26]. Epithelial-mesenchymal transition (EMT) is the process by which cancer cells acquire migratory and invasive properties, enabling them to invade surrounding tissues and metastasize. EMT is associated with enhanced tumor aggressiveness and poor prognosis [27]. Additionally, FCRL2-6, A, and B are associated with activation and inhibition of the hormone estrogen receptor (ER), which plays a crucial role in the development and progression of hormone-sensitive tumors, such as breast and endometrial cancer [28–30]. This could explain why multiple FCRL family genes significantly affect the prognosis of BRCA and UCEC in our results. FCRL1 is only associated with the activation and inhibition of the DNA damage response. The DNA damage response (DDR) is a critical mechanism for preserving genomic stability and preventing the development of cancer [31]. When the DDR pathway is activated, it can induce cell cycle arrest, DNA repair, or apoptosis. Conversely, inhibition of DDR can lead to genetic instability and the formation of tumors [31]. The FCRL gene family demonstrates the ability to activate or repress numerous critical cancer-related pathways across various cancer types. These discoveries offer valuable insights into the biological functions and potential therapeutic targets of FCRL genes in different cancer types, ultimately providing novel ideas and approaches for personalized medicine.

Our enrichment results show that FCRL family genes are primarily associated with the activation, differentiation, and proliferation of immune cells. These immune cell processes are intricately linked to tumor growth and development [32, 33], suggesting that FCRL family genes play a crucial role in regulating cancer through modulation of immunity. The GESA analysis has revealed the most common pathways enriched for FCRL family genes across various cancers. The B cell receptor (BCR) is a transmembrane receptor that recognizes and binds to specific antigens, initiating a series of events, including receptor clustering and signaling cascades, that culminate in B cell activation and differentiation [34]. The Fcγ receptor, present on the immune cell surface, binds to the Fc portion of immunoglobulins, activating downstream signaling pathways that mediate various immune responses such as phagocytosis, antibody-dependent cytotoxicity, and cytokine release [35–37]. LAT2, NTAL, and LAB play a critical role in calcium mobilization by facilitating the activation of downstream signaling molecules, resulting in the release of intracellular stores of calcium ions that promote B cell activation and proliferation [38–40]. The complement system, composed of proteins such as C4 and C2, plays a crucial role in immune defense. B cells can produce C4 and C2 activators that initiate the classical complement pathway, leading to the regulation and elimination of pathogens [41–43]. Antigen activation of the BCR triggers the production of second messengers, including phospholipase Cγ (PLCγ) [44, 45], which cleaves phosphatidylinositol 4,5-bisphosphate (PIP2) into inositol triphosphate (IP3) and diacylglycerol (DAG) [46, 47]. IP3 binds to receptors on the endoplasmic reticulum, releasing calcium, while DAG activates downstream kinases that ultimately lead to B cell activation [48, 49]. The initial complement trigger may also play a role in tumor regulation by promoting tumor cell destruction through conditioning and immune cell activation [50, 51].

The tumor microenvironment (TME) is a complex milieu in which tumor cells reside and develop, playing a crucial role in tumor progression, metastasis, and response to treatment [52]. The constant interplay between tumor cells and the TME is decisive in these processes [53]. Notably, FCRL family genes showed a significant positive correlation with ImmuneScore, StromalScore, and ESTIMATEScore in various cancers, suggesting that the FCRL family is highly expressed in the TME. As FCRL family genes are mainly expressed in immune cells, it is plausible that they exert their tumor-regulatory effects by regulating tumor-infiltrating lymphocytes within the TME.

TME comprises immune and stromal cells that play critical roles in tumor development and progression. These cells are crucial for the diagnosis and prognostic evaluation of tumors. [54]. FCRL family genes have been found to be strongly positively correlated with tumor-infiltrating lymphocytes (TILs), immunostimulators, and immunoinhibitors in pan-cancer, indicating their significant role in cancer immunomodulation. The prominent correlation with B and T cells is in line with previous GO enrichment analysis results, suggesting that FCRL family genes primarily regulate these two classes of immune cells. B cells, a vital component of TME, are essential for antitumor immunity. High expression of B cell and plasma cell signature genes and immunoglobulin levels in or around tumors is associated with a favorable prognosis in various tumor types [55]. Additionally, recent studies have demonstrated the involvement of B cells in the formation and maintenance of tertiary lymphoid structures (TLS), which are linked to protective immunity in cancer patients [56]. B cells modulate T-cell activation, expansion, and memory formation, as well as the initiation and expansion of CD4+ T cells, and the antigen cross-presentation of CD8+ T cells [55]. Furthermore, B cells activate CD4 and CD8+ T cell responses by stimulating antigen-presenting cells through immune complexes [57]. Finally, B cells have been found to enhance the survival and proliferation of cytotoxic T lymphocytes in several studies [58]. T cells exhibit the ability to differentiate between healthy and cancerous cells via antigen recognition on cell surfaces. Following recognition of cancerous cell antigens, T cells can be activated to trigger an immune response against cancerous cells [59]. This immune response involves the secretion of cytokines that recruit other immune cells to directly eliminate cancerous cells, and the development of memory T cells that offer long-lasting protection against cancer recurrence [60, 61]. T-cell-based immunotherapies are promising treatments for cancer, which involve enhancing the ability of T cells to recognize and attack cancerous cells [62].

Immunostimulators are molecules that can enhance immune cell activation and proliferation, thereby promoting an immune response against tumors. They do so by increasing the production of cytokines and chemokines that support immune-mediated destruction of tumor cells [63, 64]. The presence of immunostimulants in the tumor microenvironment is associated with better clinical outcomes across various cancers. Dysregulation of the CD70-CD27 axis in the tumor and its microenvironment is linked to tumor progression and immunosuppression [65, 66]. TNFRSF17 is a biomarker of tumor load in multiple myeloma and a target of several immunotherapies. The selection of TNFRSF17-pure deletion clones may represent a mechanism of immune escape [67]. CD48 can participate in GDF15-induced regulatory T cell generation and enhanced function, thereby regulating hepatocellular carcinoma-associated immunosuppression [68]. IL2RA is extensively involved in T cell regulation and plays a crucial role in tumor immunotherapy [69, 70].

Notably, the FCRL family genes demonstrated a significant positive correlation with several immunostimulators, particularly TIGIT, PDCD1, and BTLA, across a range of cancer types. Immune checkpoints, which are critical in regulating tumor immunoregulation, prognosis, and therapy, enable tumors to evade immune surveillance and destruction [71]. TIGIT, for instance, suppresses T cell function and controls T cell-mediated and natural killer cell-mediated tumor recognition.[72] Therefore, inhibiting TIGIT selectively may represent a rational strategy for cancer immunotherapy [73]. PD-1, or programmed cell death protein 1, is a cell surface receptor expressed on immune cells such as T cells. High levels of PD-L1 in various cancer types allow cancer cells to evade T-cell immunity through PD-L1/PD-1 signaling [74]. Blocking the PD-L1/PD-1 pathway has consistently demonstrated significant antitumor effects in patients with advanced cancers [75]. BTLA, an essential co-signaling molecule structurally and functionally similar to PD-1 and CTLA-4, is expressed in TILs and is usually associated with impaired antitumor immune responses [76]. The tumor microenvironment is a complex and dynamic ecosystem, and our findings suggest that FCRL family genes significantly regulate immune cells and components within the tumor microenvironment. Further exploration of these mechanisms may improve the efficacy of current cancer therapies and provide new therapeutic targets.

The potential role of FCRL family genes in drug resistance in tumor therapy is a critical area of investigation. Interestingly, our analysis revealed a negative correlation between FCRL family gene expression and the IC50 of various drugs in pan-cancer. These findings suggest that FCRL family genes may increase the sensitivity of these drugs in tumor therapy. Overcoming drug resistance is a significant challenge in cancer treatment [77], and a thorough exploration of the underlying mechanisms of FCRL family genes in drug resistance may provide valuable insights for the development of effective therapeutic strategies.

Our study underscores the crucial role of FCRL family genes in the pathogenesis and advancement of cancer. These genes hold great promise as therapeutic targets, especially in conjunction with immune-based therapies, to enhance the effectiveness of cancer treatment. Our findings demonstrate that targeted modulation of these genes has the potential to significantly augment the efficacy of cancer treatments. Therefore, further investigations are warranted to fully elucidate the utility of FCRL family genes as therapeutic targets in the treatment of cancer.

## MATERIALS AND METHODS

### The expression of FCRL family in 33 cancers

The mRNA expression levels of the FCRL gene family were obtained from both the TCGA and GTEx databases for 33 distinct cancer types and their corresponding normal tissue counterparts. To maintain the integrity of the data analysis, samples with gene expression levels recorded as “0” were systematically excluded. To determine any significant differences between the two groups, a Wilcoxon rank sum test was performed using the R software (version 3.6.3). The median gene expression method was employed to calculate the relevant cutoff values. The expression levels of the FCRL family were then visualized through the use of bar plots generated using the “ggplot2” package (version 3.3.3).

### Survival analysis of FCRL family in 34 cancers

The “survival” package was used to conduct Kaplan–Meier (K-M) analysis. The OS between the high- and low-FCRL family gene expression groups were compared in 34 cancers. The *p*-value was determined by Cox regression analysis. The forest plots plotted the Hazard ratio (HR), 95% Confidence Interval (CI), and *p*-value of survival curves were calculated and visualized by “survminer” and “ggplot2” (v3.3.3) package.

### Genetic alteration analysis of FCRL family

The cBioPortal (https://www.cbioportal.org/) was searched for genetic alteration information of the FCRL family gene in Pan-cancer analysis of whole genomes (ICGC/TCGA, nature 2020). “OncoPrint” module was performed to explore the genetic alterations. The mutation sites were obtained from the “mutations” module. The effect of genetic alterations on OS was explored through the “survivor” module.

### GSCALite

GSCALite [78] provides a comprehensive, scientific, and efficient analysis platform for cancer research and clinical treatment, mainly for cancer researchers and clinicians. GSCALite can integrate and analyze a large amount of genomic data such as mutations, copy number variation, transcriptomic data, epigenomic data, etc. to better understand the occurrence and progression of cancer. We have used the GSCALite platform to analyze CNV, SNV, Methylation, Pathway Activity, and Drug Sensitivity (based on the CTRP and GDSC) of FCRL family genes in pan-cancer.

### PPI network analyses of FCRL gene

The FCRL gene family was transferred into the STRING (https://string-db.org/) database, a widely utilized platform for PPI network construction. The significance criteria were rigorously established at a threshold confidence score of greater than 0.4, ensuring reliable and robust results. The resulting data were then integrated into the Cytoscape software platform (version 3.8.2) for comprehensive visualization and in-depth analysis. Utilizing the CytoHubba plugin, the top 10 nodes were determined through the application of the MCC algorithm and identified as the most prominent hub genes within the network.

### Functional enrichment analysis of FCRL family gene

We selected several cancers in which the expression of FCRL family genes significantly affected OS. The top 100 closely related genes of FCRL family genes were looked for in these cancers and then were used to perform GO function and KEGG enrichment analyses via “clusterProfiler” and “org.Hs.eg.db” packages of R software. For GO and KEGG pathway enrichment studies, the cutoff level was chosen at *p*-value < 0.01. The findings were shown as a bubble chart using “ggplot2”.

### Gene set enrichment analysis

We used R to do a “DESeq2” analysis to find different expression genes (DEGs) between high- and low-FCRL family gene groups in cancers in which expression of FCRL family genes significantly affected OS using an unpaired Student’s *t*-test. Thresholds were established at *P* < 0.05 and an absolute log-fold change greater than 1.

GSEA was done using the “clusterProfiler” package to assess the biological pathway differences between high- and low-FCRL family gene groupings. A false discovery rate (FDR) of 0.25 and an adjusted *p*-value of 0.05 were judged to be significantly altered pathways. Gene set permutation should be conducted 1,000 times for each analysis. The top ten enrichment results entries are depicted as a mountain map. The R tool “ggplot2” was used to illustrate the GSEA findings.

### Relationship between FCRL family gene and tumor immune microenvironment in pan-cancer

The “GSVA” package was used in conjunction with the “ssGSEA” algorithm to investigate the correlation between the FCRL family genes expression and markers of TILs, immunostimulators, and immunoinhibitors in 33 cancers. Additionally, we utilized the “ESTIMATE” algorithm to assess the connection between the FCRL family gene and the Stromalscore, Immune score, and ESTIMATE score of the immune matrix in cancers in which expression of FCRL family genes significantly affected OS. The connection was determined using Spearman’s correlation. To compare groups with high and low FCRL family gene expression in terms of Stromalscore, Immune score, and ESTIMATE score, the *t*-test was utilized. Statistical significance was defined as *P*-values less than 0.05. Correlations were shown as heatmaps, and the “ggplot2” package was used to compare high and low FCRL family gene expression groups. Correlations between 0–3 are considered weak, 4–6 moderate, and >7 strong.

### Data availability statement

The data that support the findings of this study are available from the corresponding author, YC.F., upon reasonable request.

## Supplementary Materials

Supplementary Figure 1

Supplementary Table 1
