# Supplementary material for: The potential of FCRL genes as targets for cancer treatment: insights from bioinformatics and immunology
Source: Aging (Albany NY). 2023 Jun 2;15(11):4926–48. doi: 10.18632/aging.204766 (PMC10292877; doi:10.18632/aging.204766)
Supplement: Supplementary Figure 1 [file aging-15-204766-s001.pdf]

SUPPLEMENTARY FIGURE

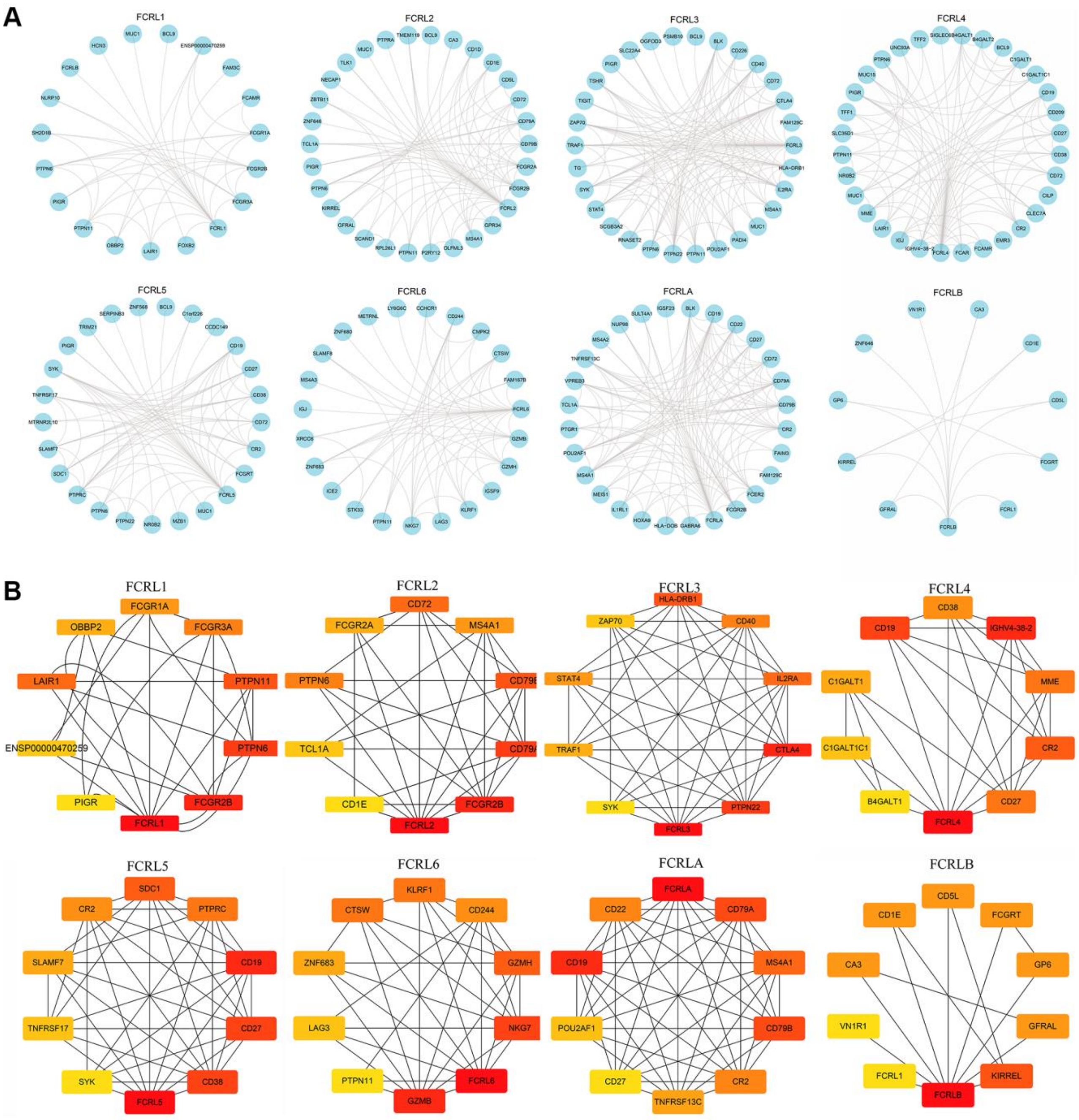

Supplementary Figure 1. The PPI network of FCRL family and related hub genes. (**A**) The PPI network of FCRL family. (**B**) The related hub genes of PPI network of FCRL family.
