# Supplementary material for: The potential of FCRL genes as targets for cancer treatment: insights from bioinformatics and immunology
Source: Aging (Albany NY). 2023 Jun 2;15(11):4926–48. doi: 10.18632/aging.204766 (PMC10292877; doi:10.18632/aging.204766)
Supplement: Supplementary Table 1 [file aging-15-204766-s002.docx]

**Supplementary Table 1. The top ten enriched terms related to the FCRL ^®^family genes expression in different cancers.**

|  |  | ID | enrichmentScore | p.adjust |
| --- | --- | --- | --- | --- |
| FCRL1 | BRCA | REACTOME_CD22_MEDIATED_BCR_REGULATION | 0.976375226 | 4.28E-09 |
|  |  | REACTOME_ROLE_OF_PHOSPHOLIPIDS_IN_PHAGOCYTOSIS | 0.945746724 | 4.28E-09 |
|  |  | REACTOME_FCGR_ACTIVATION | 0.953997567 | 4.28E-09 |
|  |  | REACTOME_ROLE_OF_LAT2_NTAL_LAB_ON_CALCIUM_MOBILIZATION | 0.947674828 | 4.28E-09 |
|  |  | REACTOME_CREATION_OF_C4_AND_C2_ACTIVATORS | 0.94743844 | 4.28E-09 |
|  |  | REACTOME_INITIAL_TRIGGERING_OF_COMPLEMENT | 0.935936941 | 4.28E-09 |
|  |  | REACTOME_SCAVENGING_OF_HEME_FROM_PLASMA | 0.944093177 | 4.28E-09 |
|  |  | REACTOME_ANTIGEN_ACTIVATES_B_CELL_RECEPTOR_BCR_LEADING_TO_GENERATION_OF_SECOND_MESSENGERS | 0.928623742 | 4.28E-09 |
|  |  | REACTOME_FCGR3A_MEDIATED_IL10_SYNTHESIS | 0.917127368 | 4.28E-09 |
|  |  | REACTOME_PARASITE_INFECTION | 0.902471034 | 4.28E-09 |
|  |  |  |  |  |
|  | CESC | REACTOME_CD22_MEDIATED_BCR_REGULATION | 0.972169932 | 6.15E-09 |
|  |  | REACTOME_FCGR_ACTIVATION | 0.951326105 | 6.15E-09 |
|  |  | REACTOME_SCAVENGING_OF_HEME_FROM_PLASMA | 0.943240931 | 6.15E-09 |
|  |  | REACTOME_ROLE_OF_LAT2_NTAL_LAB_ON_CALCIUM_MOBILIZATION | 0.939208837 | 6.15E-09 |
|  |  | REACTOME_ROLE_OF_PHOSPHOLIPIDS_IN_PHAGOCYTOSIS | 0.938453788 | 6.15E-09 |
|  |  | BIOCARTA_TCYTOTOXIC_PATHWAY | 0.938404389 | 9.01E-07 |
|  |  | BIOCARTA_THELPER_PATHWAY | 0.938314749 | 9.09E-07 |
|  |  | REACTOME_CREATION_OF_C4_AND_C2_ACTIVATORS | 0.936444042 | 6.15E-09 |
|  |  | REACTOME_ANTIGEN_ACTIVATES_B_CELL_RECEPTOR_BCR_LEADING_TO_GENERATION_OF_SECOND_MESSENGERS | 0.931563098 | 6.15E-09 |
|  |  | BIOCARTA_MONOCYTE_PATHWAY | 0.924329549 | 3.12E-05 |
|  |  |  |  |  |
|  | HNSC | REACTOME_CD22_MEDIATED_BCR_REGULATION | 0.982110042 | 4.79E-09 |
|  |  | REACTOME_SCAVENGING_OF_HEME_FROM_PLASMA | 0.955908128 | 4.79E-09 |
|  |  | REACTOME_FCGR_ACTIVATION | 0.945215582 | 4.79E-09 |
|  |  | REACTOME_CREATION_OF_C4_AND_C2_ACTIVATORS | 0.940069055 | 4.79E-09 |
|  |  | REACTOME_ROLE_OF_LAT2_NTAL_LAB_ON_CALCIUM_MOBILIZATION | 0.938787663 | 4.79E-09 |
|  |  | REACTOME_ANTIGEN_ACTIVATES_B_CELL_RECEPTOR_BCR_LEADING_TO_GENERATION_OF_SECOND_MESSENGERS | 0.937741292 | 4.79E-09 |
|  |  | REACTOME_ROLE_OF_PHOSPHOLIPIDS_IN_PHAGOCYTOSIS | 0.929109619 | 4.79E-09 |
|  |  | REACTOME_INITIAL_TRIGGERING_OF_COMPLEMENT | 0.9147497 | 4.79E-09 |
|  |  | REACTOME_FCERI_MEDIATED_MAPK_ACTIVATION | 0.904716305 | 4.79E-09 |
|  |  | REACTOME_BINDING_AND_UPTAKE_OF_LIGANDS_BY_SCAVENGER_RECEPTORS | 0.897434893 | 4.79E-09 |
|  |  |  |  |  |
|  | LIHC | REACTOME_CD22_MEDIATED_BCR_REGULATION | 0.979868684 | 3.71E-09 |
|  |  | REACTOME_SCAVENGING_OF_HEME_FROM_PLASMA | 0.970444728 | 3.71E-09 |
|  |  | REACTOME_ROLE_OF_LAT2_NTAL_LAB_ON_CALCIUM_MOBILIZATION | 0.969608579 | 3.71E-09 |
|  |  | REACTOME_CREATION_OF_C4_AND_C2_ACTIVATORS | 0.960699566 | 3.71E-09 |
|  |  | REACTOME_FCGR_ACTIVATION | 0.952956477 | 3.71E-09 |
|  |  | REACTOME_INITIAL_TRIGGERING_OF_COMPLEMENT | 0.950260573 | 3.71E-09 |
|  |  | REACTOME_ANTIGEN_ACTIVATES_B_CELL_RECEPTOR_BCR_LEADING_TO_GENERATION_OF_SECOND_MESSENGERS | 0.945402595 | 3.71E-09 |
|  |  | REACTOME_ROLE_OF_PHOSPHOLIPIDS_IN_PHAGOCYTOSIS | 0.941001527 | 3.71E-09 |
|  |  | REACTOME_FCERI_MEDIATED_MAPK_ACTIVATION | 0.938046254 | 3.71E-09 |
|  |  | REACTOME_FCERI_MEDIATED_CA_2_MOBILIZATION | 0.931303715 | 3.71E-09 |
|  |  |  |  |  |
|  | LUAD | REACTOME_CD22_MEDIATED_BCR_REGULATION | 0.859831948 | 8.6E-09 |
|  |  | REACTOME_FCGR_ACTIVATION | 0.819683385 | 8.6E-09 |
|  |  | BIOCARTA_TCYTOTOXIC_PATHWAY | 0.814577871 | 0.013691 |
|  |  | REACTOME_ROLE_OF_LAT2_NTAL_LAB_ON_CALCIUM_MOBILIZATION | 0.812363533 | 8.6E-09 |
|  |  | REACTOME_SCAVENGING_OF_HEME_FROM_PLASMA | 0.810195465 | 8.6E-09 |
|  |  | REACTOME_ROLE_OF_PHOSPHOLIPIDS_IN_PHAGOCYTOSIS | 0.803432542 | 8.6E-09 |
|  |  | REACTOME_ANTIGEN_ACTIVATES_B_CELL_RECEPTOR_BCR_LEADING_TO_GENERATION_OF_SECOND_MESSENGERS | 0.795449838 | 8.6E-09 |
|  |  | REACTOME_RUNX1_AND_FOXP3_CONTROL_THE_DEVELOPMENT_OF_REGULATORY_T_LYMPHOCYTES_TREGS | 0.792832574 | 0.068612 |
|  |  | REACTOME_FCERI_MEDIATED_CA_2_MOBILIZATION | 0.783905155 | 8.6E-09 |
|  |  | BIOCARTA_THELPER_PATHWAY | 0.782844459 | 0.032334 |
|  |  |  |  |  |
|  | READ | REACTOME_CD22_MEDIATED_BCR_REGULATION | 0.97109029 | 5.04E-09 |
|  |  | REACTOME_ROLE_OF_LAT2_NTAL_LAB_ON_CALCIUM_MOBILIZATION | 0.952140798 | 5.04E-09 |
|  |  | REACTOME_SCAVENGING_OF_HEME_FROM_PLASMA | 0.948904922 | 5.04E-09 |
|  |  | REACTOME_CREATION_OF_C4_AND_C2_ACTIVATORS | 0.946134841 | 5.04E-09 |
|  |  | REACTOME_FCGR_ACTIVATION | 0.941393753 | 5.04E-09 |
|  |  | REACTOME_INITIAL_TRIGGERING_OF_COMPLEMENT | 0.938960131 | 5.04E-09 |
|  |  | REACTOME_ROLE_OF_PHOSPHOLIPIDS_IN_PHAGOCYTOSIS | 0.929804552 | 5.04E-09 |
|  |  | REACTOME_ANTIGEN_ACTIVATES_B_CELL_RECEPTOR_BCR_LEADING_TO_GENERATION_OF_SECOND_MESSENGERS | 0.927921989 | 5.04E-09 |
|  |  | REACTOME_FCERI_MEDIATED_CA_2_MOBILIZATION | 0.926230933 | 5.04E-09 |
|  |  | REACTOME_FCERI_MEDIATED_MAPK_ACTIVATION | 0.923862055 | 5.04E-09 |
|  |  |  |  |  |
|  | SARC | REACTOME_CD22_MEDIATED_BCR_REGULATION | 0.978988849 | 6.46E-09 |
|  |  | REACTOME_SCAVENGING_OF_HEME_FROM_PLASMA | 0.971246365 | 6.46E-09 |
|  |  | REACTOME_FCGR_ACTIVATION | 0.966886619 | 6.46E-09 |
|  |  | REACTOME_ROLE_OF_LAT2_NTAL_LAB_ON_CALCIUM_MOBILIZATION | 0.965910522 | 6.46E-09 |
|  |  | REACTOME_CREATION_OF_C4_AND_C2_ACTIVATORS | 0.959666167 | 6.46E-09 |
|  |  | REACTOME_ROLE_OF_PHOSPHOLIPIDS_IN_PHAGOCYTOSIS | 0.953413583 | 6.46E-09 |
|  |  | REACTOME_FCERI_MEDIATED_MAPK_ACTIVATION | 0.944572715 | 6.46E-09 |
|  |  | REACTOME_FCERI_MEDIATED_CA_2_MOBILIZATION | 0.940766884 | 6.46E-09 |
|  |  | REACTOME_INITIAL_TRIGGERING_OF_COMPLEMENT | 0.940543409 | 6.46E-09 |
|  |  | REACTOME_FCGR3A_MEDIATED_IL10_SYNTHESIS | 0.940445468 | 6.46E-09 |
|  |  |  |  |  |
|  | SKCM | REACTOME_CD22_MEDIATED_BCR_REGULATION | 0.968347674 | 4.94E-09 |
|  |  | BIOCARTA_TCRA_PATHWAY | 0.962146824 | 2.8E-07 |
|  |  | REACTOME_PD_1_SIGNALING | 0.961007769 | 4.94E-09 |
|  |  | REACTOME_ROLE_OF_LAT2_NTAL_LAB_ON_CALCIUM_MOBILIZATION | 0.95147735 | 4.94E-09 |
|  |  | REACTOME_FCGR_ACTIVATION | 0.950012473 | 4.94E-09 |
|  |  | REACTOME_SCAVENGING_OF_HEME_FROM_PLASMA | 0.948110141 | 4.94E-09 |
|  |  | BIOCARTA_TCYTOTOXIC_PATHWAY | 0.948089611 | 4.46E-06 |
|  |  | REACTOME_ANTIGEN_ACTIVATES_B_CELL_RECEPTOR_BCR_LEADING_TO_GENERATION_OF_SECOND_MESSENGERS | 0.946890516 | 4.94E-09 |
|  |  | REACTOME_ROLE_OF_PHOSPHOLIPIDS_IN_PHAGOCYTOSIS | 0.943061453 | 4.94E-09 |
|  |  | BIOCARTA_THELPER_PATHWAY | 0.940844152 | 1.62E-05 |
|  |  |  |  |  |
| FCRL2 | BRCA | REACTOME_CD22_MEDIATED_BCR_REGULATION | 0.983644809 | 0.030415 |
|  |  | REACTOME_ROLE_OF_LAT2_NTAL_LAB_ON_CALCIUM_MOBILIZATION | 0.966700437 | 0.030415 |
|  |  | REACTOME_FCGR_ACTIVATION | 0.96618695 | 0.030415 |
|  |  | REACTOME_SCAVENGING_OF_HEME_FROM_PLASMA | 0.965072168 | 0.030415 |
|  |  | REACTOME_ROLE_OF_PHOSPHOLIPIDS_IN_PHAGOCYTOSIS | 0.959042116 | 0.030415 |
|  |  | REACTOME_CREATION_OF_C4_AND_C2_ACTIVATORS | 0.957408339 | 0.030415 |
|  |  | REACTOME_ANTIGEN_ACTIVATES_B_CELL_RECEPTOR_BCR_LEADING_TO_GENERATION_OF_SECOND_MESSENGERS | 0.945946353 | 0.030415 |
|  |  | REACTOME_INITIAL_TRIGGERING_OF_COMPLEMENT | 0.9452937 | 0.030415 |
|  |  | REACTOME_FCERI_MEDIATED_MAPK_ACTIVATION | 0.937801051 | 0.030415 |
|  |  | REACTOME_PD_1_SIGNALING | 0.937383643 | 0.030415 |
|  |  |  |  |  |
|  | CESC | REACTOME_CD22_MEDIATED_BCR_REGULATION | 0.985275312 | 0.039573 |
|  |  | REACTOME_ROLE_OF_LAT2_NTAL_LAB_ON_CALCIUM_MOBILIZATION | 0.967261695 | 0.039573 |
|  |  | REACTOME_SCAVENGING_OF_HEME_FROM_PLASMA | 0.966483172 | 0.039573 |
|  |  | REACTOME_FCGR_ACTIVATION | 0.958532018 | 0.039573 |
|  |  | REACTOME_ANTIGEN_ACTIVATES_B_CELL_RECEPTOR_BCR_LEADING_TO_GENERATION_OF_SECOND_MESSENGERS | 0.957726932 | 0.039573 |
|  |  | REACTOME_CREATION_OF_C4_AND_C2_ACTIVATORS | 0.955056387 | 0.039573 |
|  |  | REACTOME_ROLE_OF_PHOSPHOLIPIDS_IN_PHAGOCYTOSIS | 0.950825748 | 0.039573 |
|  |  | REACTOME_FCERI_MEDIATED_MAPK_ACTIVATION | 0.94111419 | 0.039573 |
|  |  | REACTOME_INITIAL_TRIGGERING_OF_COMPLEMENT | 0.936481921 | 0.039573 |
|  |  | BIOCARTA_TCYTOTOXIC_PATHWAY | 0.931778681 | 0.041184 |
|  |  |  |  |  |
|  | HNSC | REACTOME_CD22_MEDIATED_BCR_REGULATION | 0.985238356 | 0.03642 |
|  |  | REACTOME_SCAVENGING_OF_HEME_FROM_PLASMA | 0.976850049 | 0.03642 |
|  |  | REACTOME_FCGR_ACTIVATION | 0.966765203 | 0.03642 |
|  |  | REACTOME_ROLE_OF_LAT2_NTAL_LAB_ON_CALCIUM_MOBILIZATION | 0.964127004 | 0.03642 |
|  |  | REACTOME_CREATION_OF_C4_AND_C2_ACTIVATORS | 0.96061755 | 0.03642 |
|  |  | REACTOME_ANTIGEN_ACTIVATES_B_CELL_RECEPTOR_BCR_LEADING_TO_GENERATION_OF_SECOND_MESSENGERS | 0.954749426 | 0.03642 |
|  |  | REACTOME_ROLE_OF_PHOSPHOLIPIDS_IN_PHAGOCYTOSIS | 0.954074427 | 0.03642 |
|  |  | REACTOME_FCERI_MEDIATED_MAPK_ACTIVATION | 0.939222079 | 0.03642 |
|  |  | REACTOME_INITIAL_TRIGGERING_OF_COMPLEMENT | 0.937287431 | 0.03642 |
|  |  | REACTOME_BINDING_AND_UPTAKE_OF_LIGANDS_BY_SCAVENGER_RECEPTORS | 0.933601312 | 0.03642 |
|  |  |  |  |  |
|  | KIRP | REACTOME_CD22_MEDIATED_BCR_REGULATION | 0.981938555 | 0.027659 |
|  |  | REACTOME_ROLE_OF_LAT2_NTAL_LAB_ON_CALCIUM_MOBILIZATION | 0.975246914 | 0.027659 |
|  |  | REACTOME_SCAVENGING_OF_HEME_FROM_PLASMA | 0.96684852 | 0.027659 |
|  |  | REACTOME_CREATION_OF_C4_AND_C2_ACTIVATORS | 0.964596878 | 0.027659 |
|  |  | REACTOME_FCGR_ACTIVATION | 0.961729712 | 0.027659 |
|  |  | REACTOME_ROLE_OF_PHOSPHOLIPIDS_IN_PHAGOCYTOSIS | 0.960608415 | 0.027659 |
|  |  | REACTOME_FCERI_MEDIATED_MAPK_ACTIVATION | 0.960193456 | 0.027659 |
|  |  | REACTOME_ANTIGEN_ACTIVATES_B_CELL_RECEPTOR_BCR_LEADING_TO_GENERATION_OF_SECOND_MESSENGERS | 0.958225006 | 0.027659 |
|  |  | REACTOME_FCERI_MEDIATED_CA_2_MOBILIZATION | 0.949794378 | 0.027659 |
|  |  | REACTOME_INITIAL_TRIGGERING_OF_COMPLEMENT | 0.947706846 | 0.027659 |
|  |  |  |  |  |
|  | LUAD | REACTOME_CD22_MEDIATED_BCR_REGULATION | 0.936031995 | 0.096486 |
|  |  | REACTOME_ROLE_OF_LAT2_NTAL_LAB_ON_CALCIUM_MOBILIZATION | 0.925549487 | 0.096486 |
|  |  | REACTOME_CREATION_OF_C4_AND_C2_ACTIVATORS | 0.920928976 | 0.096486 |
|  |  | REACTOME_FCGR_ACTIVATION | 0.916016126 | 0.096486 |
|  |  | REACTOME_SCAVENGING_OF_HEME_FROM_PLASMA | 0.913787264 | 0.096486 |
|  |  | REACTOME_INITIAL_TRIGGERING_OF_COMPLEMENT | 0.904111572 | 0.096486 |
|  |  | REACTOME_ROLE_OF_PHOSPHOLIPIDS_IN_PHAGOCYTOSIS | 0.898070339 | 0.096486 |
|  |  | REACTOME_ANTIGEN_ACTIVATES_B_CELL_RECEPTOR_BCR_LEADING_TO_GENERATION_OF_SECOND_MESSENGERS | 0.889399444 | 0.096486 |
|  |  | REACTOME_FCERI_MEDIATED_MAPK_ACTIVATION | 0.886435865 | 0.096486 |
|  |  | REACTOME_FCERI_MEDIATED_CA_2_MOBILIZATION | 0.873504813 | 0.096486 |
|  |  |  |  |  |
|  | OV | REACTOME_CD22_MEDIATED_BCR_REGULATION | 0.983127599 | 0.036648 |
|  |  | REACTOME_SCAVENGING_OF_HEME_FROM_PLASMA | 0.973035216 | 0.036648 |
|  |  | REACTOME_ROLE_OF_LAT2_NTAL_LAB_ON_CALCIUM_MOBILIZATION | 0.970942667 | 0.036648 |
|  |  | REACTOME_FCGR_ACTIVATION | 0.967878163 | 0.036648 |
|  |  | REACTOME_ROLE_OF_PHOSPHOLIPIDS_IN_PHAGOCYTOSIS | 0.966280186 | 0.036648 |
|  |  | REACTOME_CREATION_OF_C4_AND_C2_ACTIVATORS | 0.962301449 | 0.036648 |
|  |  | REACTOME_ANTIGEN_ACTIVATES_B_CELL_RECEPTOR_BCR_LEADING_TO_GENERATION_OF_SECOND_MESSENGERS | 0.960336276 | 0.036648 |
|  |  | REACTOME_FCERI_MEDIATED_MAPK_ACTIVATION | 0.953793768 | 0.036648 |
|  |  | REACTOME_FCERI_MEDIATED_CA_2_MOBILIZATION | 0.949811641 | 0.036648 |
|  |  | REACTOME_FCERI_MEDIATED_NF_KB_ACTIVATION | 0.946689896 | 0.036648 |
|  |  |  |  |  |
|  | SARC | REACTOME_CD22_MEDIATED_BCR_REGULATION | 0.984230771 | 0.037175 |
|  |  | REACTOME_SCAVENGING_OF_HEME_FROM_PLASMA | 0.976700279 | 0.037175 |
|  |  | REACTOME_CREATION_OF_C4_AND_C2_ACTIVATORS | 0.97394973 | 0.037175 |
|  |  | REACTOME_ROLE_OF_LAT2_NTAL_LAB_ON_CALCIUM_MOBILIZATION | 0.971482131 | 0.037175 |
|  |  | REACTOME_FCGR_ACTIVATION | 0.971213837 | 0.037175 |
|  |  | REACTOME_ROLE_OF_PHOSPHOLIPIDS_IN_PHAGOCYTOSIS | 0.965546776 | 0.037175 |
|  |  | REACTOME_INITIAL_TRIGGERING_OF_COMPLEMENT | 0.958574029 | 0.037175 |
|  |  | REACTOME_FCERI_MEDIATED_MAPK_ACTIVATION | 0.953986159 | 0.037175 |
|  |  | REACTOME_FCGR3A_MEDIATED_IL10_SYNTHESIS | 0.953376975 | 0.037175 |
|  |  | REACTOME_ANTIGEN_ACTIVATES_B_CELL_RECEPTOR_BCR_LEADING_TO_GENERATION_OF_SECOND_MESSENGERS | 0.951311591 | 0.037175 |
|  |  |  |  |  |
|  | SKCM | REACTOME_CD22_MEDIATED_BCR_REGULATION | 0.969929299 | 0.034961 |
|  |  | REACTOME_ROLE_OF_LAT2_NTAL_LAB_ON_CALCIUM_MOBILIZATION | 0.968192418 | 0.034961 |
|  |  | REACTOME_SCAVENGING_OF_HEME_FROM_PLASMA | 0.962133174 | 0.034961 |
|  |  | REACTOME_PD_1_SIGNALING | 0.961199562 | 0.034961 |
|  |  | BIOCARTA_TCRA_PATHWAY | 0.960758338 | 0.034961 |
|  |  | REACTOME_FCGR_ACTIVATION | 0.955208521 | 0.034961 |
|  |  | REACTOME_ANTIGEN_ACTIVATES_B_CELL_RECEPTOR_BCR_LEADING_TO_GENERATION_OF_SECOND_MESSENGERS | 0.948621374 | 0.034961 |
|  |  | BIOCARTA_TCYTOTOXIC_PATHWAY | 0.946866057 | 0.034961 |
|  |  | REACTOME_ROLE_OF_PHOSPHOLIPIDS_IN_PHAGOCYTOSIS | 0.945611649 | 0.034961 |
|  |  | REACTOME_CREATION_OF_C4_AND_C2_ACTIVATORS | 0.942220001 | 0.034961 |
|  |  |  |  |  |
| FCRL3 | BRCA | REACTOME_CD22_MEDIATED_BCR_REGULATION | 0.973536717 | 4E-09 |
|  |  | BIOCARTA_TCRA_PATHWAY | 0.951085295 | 3.6E-06 |
|  |  | BIOCARTA_CTLA4_PATHWAY | 0.950508834 | 4E-09 |
|  |  | REACTOME_FCGR_ACTIVATION | 0.949776031 | 4E-09 |
|  |  | REACTOME_PD_1_SIGNALING | 0.94845237 | 4E-09 |
|  |  | REACTOME_ROLE_OF_PHOSPHOLIPIDS_IN_PHAGOCYTOSIS | 0.945565167 | 4E-09 |
|  |  | REACTOME_ROLE_OF_LAT2_NTAL_LAB_ON_CALCIUM_MOBILIZATION | 0.940234195 | 4E-09 |
|  |  | REACTOME_CREATION_OF_C4_AND_C2_ACTIVATORS | 0.938426094 | 4E-09 |
|  |  | BIOCARTA_TCYTOTOXIC_PATHWAY | 0.937607362 | 1.81E-05 |
|  |  | BIOCARTA_THELPER_PATHWAY | 0.936495417 | 2.06E-05 |
|  |  |  |  |  |
|  | CESC | REACTOME_CD22_MEDIATED_BCR_REGULATION | 0.977467015 | 2.68E-09 |
|  |  | REACTOME_FCGR_ACTIVATION | 0.945656779 | 2.68E-09 |
|  |  | REACTOME_SCAVENGING_OF_HEME_FROM_PLASMA | 0.944677798 | 2.68E-09 |
|  |  | REACTOME_CREATION_OF_C4_AND_C2_ACTIVATORS | 0.940279693 | 2.68E-09 |
|  |  | REACTOME_ROLE_OF_PHOSPHOLIPIDS_IN_PHAGOCYTOSIS | 0.935293513 | 2.68E-09 |
|  |  | REACTOME_ROLE_OF_LAT2_NTAL_LAB_ON_CALCIUM_MOBILIZATION | 0.930814798 | 2.68E-09 |
|  |  | REACTOME_INITIAL_TRIGGERING_OF_COMPLEMENT | 0.927981779 | 2.68E-09 |
|  |  | REACTOME_ANTIGEN_ACTIVATES_B_CELL_RECEPTOR_BCR_LEADING_TO_GENERATION_OF_SECOND_MESSENGERS | 0.924976295 | 2.68E-09 |
|  |  | BIOCARTA_TCRA_PATHWAY | 0.921227644 | 2.89E-06 |
|  |  | REACTOME_FCERI_MEDIATED_CA_2_MOBILIZATION | 0.918105982 | 2.68E-09 |
|  |  |  |  |  |
|  | HNSC | REACTOME_CD22_MEDIATED_BCR_REGULATION | 0.970634698 | 4.13E-09 |
|  |  | REACTOME_FCGR_ACTIVATION | 0.949236866 | 4.13E-09 |
|  |  | REACTOME_ROLE_OF_LAT2_NTAL_LAB_ON_CALCIUM_MOBILIZATION | 0.94699169 | 4.13E-09 |
|  |  | REACTOME_CREATION_OF_C4_AND_C2_ACTIVATORS | 0.927913174 | 4.13E-09 |
|  |  | REACTOME_ROLE_OF_PHOSPHOLIPIDS_IN_PHAGOCYTOSIS | 0.920140978 | 4.13E-09 |
|  |  | REACTOME_ANTIGEN_ACTIVATES_B_CELL_RECEPTOR_BCR_LEADING_TO_GENERATION_OF_SECOND_MESSENGERS | 0.91439102 | 4.13E-09 |
|  |  | REACTOME_INITIAL_TRIGGERING_OF_COMPLEMENT | 0.912818153 | 4.13E-09 |
|  |  | REACTOME_FCERI_MEDIATED_MAPK_ACTIVATION | 0.91164805 | 4.13E-09 |
|  |  | BIOCARTA_TCRA_PATHWAY | 0.907734016 | 1.44E-05 |
|  |  | REACTOME_FCERI_MEDIATED_CA_2_MOBILIZATION | 0.902091015 | 4.13E-09 |
|  |  |  |  |  |
|  | KIRC | REACTOME_CD22_MEDIATED_BCR_REGULATION | 0.970634698 | 4.13E-09 |
|  |  | REACTOME_FCGR_ACTIVATION | 0.949236866 | 4.13E-09 |
|  |  | REACTOME_ROLE_OF_LAT2_NTAL_LAB_ON_CALCIUM_MOBILIZATION | 0.94699169 | 4.13E-09 |
|  |  | REACTOME_CREATION_OF_C4_AND_C2_ACTIVATORS | 0.927913174 | 4.13E-09 |
|  |  | REACTOME_ROLE_OF_PHOSPHOLIPIDS_IN_PHAGOCYTOSIS | 0.920140978 | 4.13E-09 |
|  |  | REACTOME_ANTIGEN_ACTIVATES_B_CELL_RECEPTOR_BCR_LEADING_TO_GENERATION_OF_SECOND_MESSENGERS | 0.91439102 | 4.13E-09 |
|  |  | REACTOME_INITIAL_TRIGGERING_OF_COMPLEMENT | 0.912818153 | 4.13E-09 |
|  |  | REACTOME_FCERI_MEDIATED_MAPK_ACTIVATION | 0.91164805 | 4.13E-09 |
|  |  | BIOCARTA_TCRA_PATHWAY | 0.907734016 | 1.44E-05 |
|  |  | REACTOME_FCERI_MEDIATED_CA_2_MOBILIZATION | 0.902091015 | 4.13E-09 |
|  |  |  |  |  |
|  | KIRP | REACTOME_CD22_MEDIATED_BCR_REGULATION | 0.981862696 | 5.48E-09 |
|  |  | REACTOME_ROLE_OF_LAT2_NTAL_LAB_ON_CALCIUM_MOBILIZATION | 0.971660305 | 5.48E-09 |
|  |  | REACTOME_SCAVENGING_OF_HEME_FROM_PLASMA | 0.969432448 | 5.48E-09 |
|  |  | REACTOME_FCGR_ACTIVATION | 0.957721615 | 5.48E-09 |
|  |  | REACTOME_FCERI_MEDIATED_MAPK_ACTIVATION | 0.953831509 | 5.48E-09 |
|  |  | REACTOME_ANTIGEN_ACTIVATES_B_CELL_RECEPTOR_BCR_LEADING_TO_GENERATION_OF_SECOND_MESSENGERS | 0.952176348 | 5.48E-09 |
|  |  | REACTOME_ROLE_OF_PHOSPHOLIPIDS_IN_PHAGOCYTOSIS | 0.95182368 | 5.48E-09 |
|  |  | REACTOME_CREATION_OF_C4_AND_C2_ACTIVATORS | 0.948622464 | 5.48E-09 |
|  |  | REACTOME_FCERI_MEDIATED_CA_2_MOBILIZATION | 0.933526576 | 5.48E-09 |
|  |  | REACTOME_FCERI_MEDIATED_NF_KB_ACTIVATION | 0.93072871 | 5.48E-09 |
|  |  |  |  |  |
|  | LIHC | REACTOME_CD22_MEDIATED_BCR_REGULATION | 0.98101256 | 5.44E-09 |
|  |  | REACTOME_SCAVENGING_OF_HEME_FROM_PLASMA | 0.975600088 | 5.44E-09 |
|  |  | REACTOME_ROLE_OF_LAT2_NTAL_LAB_ON_CALCIUM_MOBILIZATION | 0.967318408 | 5.44E-09 |
|  |  | REACTOME_FCGR_ACTIVATION | 0.958100896 | 5.44E-09 |
|  |  | REACTOME_CREATION_OF_C4_AND_C2_ACTIVATORS | 0.956887078 | 5.44E-09 |
|  |  | REACTOME_ROLE_OF_PHOSPHOLIPIDS_IN_PHAGOCYTOSIS | 0.95531423 | 5.44E-09 |
|  |  | BIOCARTA_CTLA4_PATHWAY | 0.948399309 | 1.79E-08 |
|  |  | REACTOME_INITIAL_TRIGGERING_OF_COMPLEMENT | 0.946520826 | 5.44E-09 |
|  |  | REACTOME_FCERI_MEDIATED_MAPK_ACTIVATION | 0.944562015 | 5.44E-09 |
|  |  | REACTOME_ANTIGEN_ACTIVATES_B_CELL_RECEPTOR_BCR_LEADING_TO_GENERATION_OF_SECOND_MESSENGERS | 0.943127259 | 5.44E-09 |
|  |  |  |  |  |
|  | LUAD | REACTOME_CD22_MEDIATED_BCR_REGULATION | 0.85829318 | 4.85E-09 |
|  |  | REACTOME_RUNX1_AND_FOXP3_CONTROL_THE_DEVELOPMENT_OF_REGULATORY_T_LYMPHOCYTES_TREGS | 0.848944912 | 0.006898 |
|  |  | BIOCARTA_TCYTOTOXIC_PATHWAY | 0.847294174 | 0.002063 |
|  |  | BIOCARTA_THELPER_PATHWAY | 0.842847959 | 0.002483 |
|  |  | BIOCARTA_TCRA_PATHWAY | 0.823765348 | 0.005173 |
|  |  | REACTOME_FCGR_ACTIVATION | 0.813184873 | 4.85E-09 |
|  |  | REACTOME_ROLE_OF_LAT2_NTAL_LAB_ON_CALCIUM_MOBILIZATION | 0.801914976 | 4.85E-09 |
|  |  | REACTOME_BUTYROPHILIN_BTN_FAMILY_INTERACTIONS | 0.798729541 | 0.013176 |
|  |  | REACTOME_CREATION_OF_C4_AND_C2_ACTIVATORS | 0.798143957 | 4.85E-09 |
|  |  | REACTOME_ANTIGEN_ACTIVATES_B_CELL_RECEPTOR_BCR_LEADING_TO_GENERATION_OF_SECOND_MESSENGERS | 0.796958974 | 4.85E-09 |
|  |  |  |  |  |
|  | OSCC | REACTOME_CD22_MEDIATED_BCR_REGULATION | 0.974706026 | 3.5E-09 |
|  |  | REACTOME_FCGR_ACTIVATION | 0.950618431 | 3.5E-09 |
|  |  | REACTOME_CREATION_OF_C4_AND_C2_ACTIVATORS | 0.942296108 | 3.5E-09 |
|  |  | REACTOME_ROLE_OF_PHOSPHOLIPIDS_IN_PHAGOCYTOSIS | 0.941927277 | 3.5E-09 |
|  |  | REACTOME_SCAVENGING_OF_HEME_FROM_PLASMA | 0.941789622 | 3.5E-09 |
|  |  | REACTOME_ROLE_OF_LAT2_NTAL_LAB_ON_CALCIUM_MOBILIZATION | 0.927855387 | 3.5E-09 |
|  |  | BIOCARTA_TCRA_PATHWAY | 0.924048436 | 4.56E-06 |
|  |  | REACTOME_FCERI_MEDIATED_CA_2_MOBILIZATION | 0.922537343 | 3.5E-09 |
|  |  | REACTOME_ANTIGEN_ACTIVATES_B_CELL_RECEPTOR_BCR_LEADING_TO_GENERATION_OF_SECOND_MESSENGERS | 0.919946505 | 3.5E-09 |
|  |  | REACTOME_INITIAL_TRIGGERING_OF_COMPLEMENT | 0.919445471 | 3.5E-09 |
|  |  |  |  |  |
|  | OV | REACTOME_UNWINDING_OF_DNA | 0.920317435 | 8.23E-06 |
|  |  | REACTOME_POLO_LIKE_KINASE_MEDIATED_EVENTS | 0.901522472 | 2.04E-06 |
|  |  | REACTOME_CD22_MEDIATED_BCR_REGULATION | 0.869337693 | 0.000652 |
|  |  | REACTOME_G1_S_SPECIFIC_TRANSCRIPTION | 0.855654082 | 7.58E-09 |
|  |  | REACTOME_CONDENSATION_OF_PROMETAPHASE_CHROMOSOMES | 0.854613968 | 0.001187 |
|  |  | WP_GASTRIC_CANCER_NETWORK_1 | 0.843323359 | 2.61E-07 |
|  |  | WP_REGULATION_OF_SISTER_CHROMATID_SEPARATION_AT_THE_METAPHASEANAPHASE_TRANSITION | 0.841738502 | 8.03E-05 |
|  |  | REACTOME_ACTIVATION_OF_THE_PRE_REPLICATIVE_COMPLEX | 0.841664115 | 7.58E-09 |
|  |  | BIOCARTA_TCYTOTOXIC_PATHWAY | 0.832269645 | 0.0004 |
|  |  | REACTOME_G0_AND_EARLY_G1 | 0.829708143 | 1.06E-07 |
|  |  |  |  |  |
|  | SARC | REACTOME_CD22_MEDIATED_BCR_REGULATION | 0.977232168 | 5.04E-09 |
|  |  | REACTOME_SCAVENGING_OF_HEME_FROM_PLASMA | 0.972856637 | 5.04E-09 |
|  |  | REACTOME_FCGR_ACTIVATION | 0.967479457 | 5.04E-09 |
|  |  | REACTOME_ROLE_OF_LAT2_NTAL_LAB_ON_CALCIUM_MOBILIZATION | 0.967164513 | 5.04E-09 |
|  |  | REACTOME_ROLE_OF_PHOSPHOLIPIDS_IN_PHAGOCYTOSIS | 0.966105345 | 5.04E-09 |
|  |  | REACTOME_CREATION_OF_C4_AND_C2_ACTIVATORS | 0.962963983 | 5.04E-09 |
|  |  | REACTOME_FCERI_MEDIATED_MAPK_ACTIVATION | 0.947666381 | 5.04E-09 |
|  |  | REACTOME_PD_1_SIGNALING | 0.947136231 | 5.04E-09 |
|  |  | REACTOME_ANTIGEN_ACTIVATES_B_CELL_RECEPTOR_BCR_LEADING_TO_GENERATION_OF_SECOND_MESSENGERS | 0.946124171 | 5.04E-09 |
|  |  | REACTOME_BINDING_AND_UPTAKE_OF_LIGANDS_BY_SCAVENGER_RECEPTORS | 0.945793593 | 5.04E-09 |
|  |  |  |  |  |
|  | SKCM | REACTOME_UNWINDING_OF_DNA | 0.920317435 | 8.23E-06 |
|  |  | REACTOME_POLO_LIKE_KINASE_MEDIATED_EVENTS | 0.901522472 | 2.04E-06 |
|  |  | REACTOME_CD22_MEDIATED_BCR_REGULATION | 0.869337693 | 0.000652 |
|  |  | REACTOME_G1_S_SPECIFIC_TRANSCRIPTION | 0.855654082 | 7.58E-09 |
|  |  | REACTOME_CONDENSATION_OF_PROMETAPHASE_CHROMOSOMES | 0.854613968 | 0.001187 |
|  |  | WP_GASTRIC_CANCER_NETWORK_1 | 0.843323359 | 2.61E-07 |
|  |  | WP_REGULATION_OF_SISTER_CHROMATID_SEPARATION_AT_THE_METAPHASEANAPHASE_TRANSITION | 0.841738502 | 8.03E-05 |
|  |  | REACTOME_ACTIVATION_OF_THE_PRE_REPLICATIVE_COMPLEX | 0.841664115 | 7.58E-09 |
|  |  | BIOCARTA_TCYTOTOXIC_PATHWAY | 0.832269645 | 0.0004 |
|  |  | REACTOME_G0_AND_EARLY_G1 | 0.829708143 | 1.06E-07 |
|  |  |  |  |  |
|  | UCEC | REACTOME_CD22_MEDIATED_BCR_REGULATION | 0.979708212 | 4.49E-09 |
|  |  | REACTOME_FCGR_ACTIVATION | 0.962732454 | 4.49E-09 |
|  |  | REACTOME_ROLE_OF_LAT2_NTAL_LAB_ON_CALCIUM_MOBILIZATION | 0.961238129 | 4.49E-09 |
|  |  | REACTOME_CREATION_OF_C4_AND_C2_ACTIVATORS | 0.956352031 | 4.49E-09 |
|  |  | REACTOME_SCAVENGING_OF_HEME_FROM_PLASMA | 0.955918918 | 4.49E-09 |
|  |  | REACTOME_ROLE_OF_PHOSPHOLIPIDS_IN_PHAGOCYTOSIS | 0.954517568 | 4.49E-09 |
|  |  | REACTOME_FCERI_MEDIATED_CA_2_MOBILIZATION | 0.951466924 | 4.49E-09 |
|  |  | REACTOME_FCERI_MEDIATED_MAPK_ACTIVATION | 0.948524983 | 4.49E-09 |
|  |  | REACTOME_ANTIGEN_ACTIVATES_B_CELL_RECEPTOR_BCR_LEADING_TO_GENERATION_OF_SECOND_MESSENGERS | 0.944142948 | 4.49E-09 |
|  |  | BIOCARTA_TCYTOTOXIC_PATHWAY | 0.943345199 | 1.49E-05 |
|  |  |  |  |  |
|  | UVM | BIOCARTA_TCRA_PATHWAY | 0.941549831 | 5.98E-07 |
|  |  | REACTOME_CD22_MEDIATED_BCR_REGULATION | 0.935259489 | 2.68E-09 |
|  |  | REACTOME_SCAVENGING_OF_HEME_FROM_PLASMA | 0.934332531 | 2.68E-09 |
|  |  | REACTOME_FCGR_ACTIVATION | 0.934309181 | 2.68E-09 |
|  |  | REACTOME_ROLE_OF_PHOSPHOLIPIDS_IN_PHAGOCYTOSIS | 0.92530524 | 2.68E-09 |
|  |  | BIOCARTA_CTLA4_PATHWAY | 0.924787763 | 2.68E-09 |
|  |  | BIOCARTA_CTL_PATHWAY | 0.917068265 | 2.63E-06 |
|  |  | REACTOME_CREATION_OF_C4_AND_C2_ACTIVATORS | 0.917002075 | 2.68E-09 |
|  |  | KEGG_GRAFT_VERSUS_HOST_DISEASE | 0.916067813 | 2.68E-09 |
|  |  | BIOCARTA_TCYTOTOXIC_PATHWAY | 0.915228468 | 2.15E-05 |
|  |  |  |  |  |
| FCRL4 | LUAD | REACTOME_CD22_MEDIATED_BCR_REGULATION | 0.9757926 | 5.32E-09 |
|  |  | REACTOME_ROLE_OF_LAT2_NTAL_LAB_ON_CALCIUM_MOBILIZATION | 0.961249479 | 5.32E-09 |
|  |  | REACTOME_CREATION_OF_C4_AND_C2_ACTIVATORS | 0.95121899 | 5.32E-09 |
|  |  | REACTOME_SCAVENGING_OF_HEME_FROM_PLASMA | 0.944988284 | 5.32E-09 |
|  |  | REACTOME_FCGR_ACTIVATION | 0.941989261 | 5.32E-09 |
|  |  | REACTOME_INITIAL_TRIGGERING_OF_COMPLEMENT | 0.939820764 | 5.32E-09 |
|  |  | REACTOME_ROLE_OF_PHOSPHOLIPIDS_IN_PHAGOCYTOSIS | 0.924623189 | 5.32E-09 |
|  |  | REACTOME_ANTIGEN_ACTIVATES_B_CELL_RECEPTOR_BCR_LEADING_TO_GENERATION_OF_SECOND_MESSENGERS | 0.924126919 | 5.32E-09 |
|  |  | REACTOME_FCERI_MEDIATED_MAPK_ACTIVATION | 0.919680072 | 5.32E-09 |
|  |  | REACTOME_FCERI_MEDIATED_CA_2_MOBILIZATION | 0.903373161 | 5.32E-09 |
|  |  |  |  |  |
|  | HNSC | REACTOME_CD22_MEDIATED_BCR_REGULATION | 0.982501161 | 4.28E-09 |
|  |  | REACTOME_CREATION_OF_C4_AND_C2_ACTIVATORS | 0.958551445 | 4.28E-09 |
|  |  | REACTOME_SCAVENGING_OF_HEME_FROM_PLASMA | 0.958122072 | 4.28E-09 |
|  |  | REACTOME_ROLE_OF_LAT2_NTAL_LAB_ON_CALCIUM_MOBILIZATION | 0.952833265 | 4.28E-09 |
|  |  | REACTOME_ANTIGEN_ACTIVATES_B_CELL_RECEPTOR_BCR_LEADING_TO_GENERATION_OF_SECOND_MESSENGERS | 0.945114764 | 4.28E-09 |
|  |  | REACTOME_FCGR_ACTIVATION | 0.94453399 | 4.28E-09 |
|  |  | REACTOME_ROLE_OF_PHOSPHOLIPIDS_IN_PHAGOCYTOSIS | 0.938551671 | 4.28E-09 |
|  |  | REACTOME_INITIAL_TRIGGERING_OF_COMPLEMENT | 0.923821509 | 4.28E-09 |
|  |  | REACTOME_FCERI_MEDIATED_MAPK_ACTIVATION | 0.920834518 | 4.28E-09 |
|  |  | REACTOME_BINDING_AND_UPTAKE_OF_LIGANDS_BY_SCAVENGER_RECEPTORS | 0.90951939 | 4.28E-09 |
|  |  |  |  |  |
|  | SKCM | REACTOME_CD22_MEDIATED_BCR_REGULATION | 0.971648585 | 6.15E-09 |
|  |  | REACTOME_ROLE_OF_LAT2_NTAL_LAB_ON_CALCIUM_MOBILIZATION | 0.965104173 | 6.15E-09 |
|  |  | REACTOME_SCAVENGING_OF_HEME_FROM_PLASMA | 0.952431133 | 6.15E-09 |
|  |  | REACTOME_FCGR_ACTIVATION | 0.947696254 | 6.15E-09 |
|  |  | REACTOME_ANTIGEN_ACTIVATES_B_CELL_RECEPTOR_BCR_LEADING_TO_GENERATION_OF_SECOND_MESSENGERS | 0.945925305 | 6.15E-09 |
|  |  | REACTOME_ROLE_OF_PHOSPHOLIPIDS_IN_PHAGOCYTOSIS | 0.94392656 | 6.15E-09 |
|  |  | BIOCARTA_TCYTOTOXIC_PATHWAY | 0.936068726 | 8.57E-05 |
|  |  | REACTOME_PD_1_SIGNALING | 0.934937219 | 6.15E-09 |
|  |  | BIOCARTA_TCRA_PATHWAY | 0.933783676 | 0.000114 |
|  |  | REACTOME_FCERI_MEDIATED_MAPK_ACTIVATION | 0.933298678 | 6.15E-09 |
|  |  |  |  |  |
| FCRL5 | BRCA | REACTOME_CD22_MEDIATED_BCR_REGULATION | 0.982948143 | 5.59E-09 |
|  |  | REACTOME_ROLE_OF_LAT2_NTAL_LAB_ON_CALCIUM_MOBILIZATION | 0.971114171 | 5.59E-09 |
|  |  | REACTOME_FCGR_ACTIVATION | 0.967023158 | 5.59E-09 |
|  |  | REACTOME_SCAVENGING_OF_HEME_FROM_PLASMA | 0.966305086 | 5.59E-09 |
|  |  | REACTOME_CREATION_OF_C4_AND_C2_ACTIVATORS | 0.962481289 | 5.59E-09 |
|  |  | REACTOME_ROLE_OF_PHOSPHOLIPIDS_IN_PHAGOCYTOSIS | 0.959139181 | 5.59E-09 |
|  |  | REACTOME_ANTIGEN_ACTIVATES_B_CELL_RECEPTOR_BCR_LEADING_TO_GENERATION_OF_SECOND_MESSENGERS | 0.948348763 | 5.59E-09 |
|  |  | REACTOME_FCERI_MEDIATED_MAPK_ACTIVATION | 0.947271464 | 5.59E-09 |
|  |  | REACTOME_INITIAL_TRIGGERING_OF_COMPLEMENT | 0.945873863 | 5.59E-09 |
|  |  | REACTOME_FCGR3A_MEDIATED_IL10_SYNTHESIS | 0.940165247 | 5.59E-09 |
|  |  |  |  |  |
|  | CESC | REACTOME_CD22_MEDIATED_BCR_REGULATION | 0.986862738 | 7.41E-09 |
|  |  | REACTOME_SCAVENGING_OF_HEME_FROM_PLASMA | 0.975518164 | 7.41E-09 |
|  |  | REACTOME_ROLE_OF_LAT2_NTAL_LAB_ON_CALCIUM_MOBILIZATION | 0.972066574 | 7.41E-09 |
|  |  | REACTOME_FCGR_ACTIVATION | 0.962862541 | 7.41E-09 |
|  |  | REACTOME_ANTIGEN_ACTIVATES_B_CELL_RECEPTOR_BCR_LEADING_TO_GENERATION_OF_SECOND_MESSENGERS | 0.960709673 | 7.41E-09 |
|  |  | REACTOME_ROLE_OF_PHOSPHOLIPIDS_IN_PHAGOCYTOSIS | 0.958179372 | 7.41E-09 |
|  |  | REACTOME_FCERI_MEDIATED_MAPK_ACTIVATION | 0.952521563 | 7.41E-09 |
|  |  | REACTOME_CREATION_OF_C4_AND_C2_ACTIVATORS | 0.951296867 | 7.41E-09 |
|  |  | REACTOME_FCERI_MEDIATED_CA_2_MOBILIZATION | 0.943902602 | 7.41E-09 |
|  |  | REACTOME_FCGR3A_MEDIATED_IL10_SYNTHESIS | 0.939251247 | 7.41E-09 |
|  |  |  |  |  |
|  | COAD | REACTOME_CD22_MEDIATED_BCR_REGULATION | 0.960342434 | 8.17E-09 |
|  |  | REACTOME_SCAVENGING_OF_HEME_FROM_PLASMA | 0.947799616 | 8.17E-09 |
|  |  | REACTOME_ROLE_OF_LAT2_NTAL_LAB_ON_CALCIUM_MOBILIZATION | 0.940202734 | 8.17E-09 |
|  |  | REACTOME_CREATION_OF_C4_AND_C2_ACTIVATORS | 0.938789523 | 8.17E-09 |
|  |  | REACTOME_FCGR_ACTIVATION | 0.933885114 | 8.17E-09 |
|  |  | REACTOME_ROLE_OF_PHOSPHOLIPIDS_IN_PHAGOCYTOSIS | 0.918537924 | 8.17E-09 |
|  |  | REACTOME_INITIAL_TRIGGERING_OF_COMPLEMENT | 0.910641192 | 8.17E-09 |
|  |  | REACTOME_FCERI_MEDIATED_MAPK_ACTIVATION | 0.908974609 | 8.17E-09 |
|  |  | REACTOME_ANTIGEN_ACTIVATES_B_CELL_RECEPTOR_BCR_LEADING_TO_GENERATION_OF_SECOND_MESSENGERS | 0.906213895 | 8.17E-09 |
|  |  | REACTOME_FCERI_MEDIATED_CA_2_MOBILIZATION | 0.88947843 | 8.17E-09 |
|  |  |  |  |  |
|  | HNSC | REACTOME_CD22_MEDIATED_BCR_REGULATION | 0.986849218 | 2.9E-09 |
|  |  | REACTOME_SCAVENGING_OF_HEME_FROM_PLASMA | 0.974237624 | 2.9E-09 |
|  |  | REACTOME_FCGR_ACTIVATION | 0.972387117 | 2.9E-09 |
|  |  | REACTOME_ROLE_OF_LAT2_NTAL_LAB_ON_CALCIUM_MOBILIZATION | 0.970005834 | 2.9E-09 |
|  |  | REACTOME_CREATION_OF_C4_AND_C2_ACTIVATORS | 0.967443997 | 2.9E-09 |
|  |  | REACTOME_ROLE_OF_PHOSPHOLIPIDS_IN_PHAGOCYTOSIS | 0.961918544 | 2.9E-09 |
|  |  | REACTOME_ANTIGEN_ACTIVATES_B_CELL_RECEPTOR_BCR_LEADING_TO_GENERATION_OF_SECOND_MESSENGERS | 0.959798512 | 2.9E-09 |
|  |  | REACTOME_FCERI_MEDIATED_MAPK_ACTIVATION | 0.951004833 | 2.9E-09 |
|  |  | REACTOME_INITIAL_TRIGGERING_OF_COMPLEMENT | 0.943485061 | 2.9E-09 |
|  |  | REACTOME_BINDING_AND_UPTAKE_OF_LIGANDS_BY_SCAVENGER_RECEPTORS | 0.941837922 | 2.9E-09 |
|  |  |  |  |  |
|  | KIRC | REACTOME_CD22_MEDIATED_BCR_REGULATION | 0.989905311 | 5.8E-09 |
|  |  | REACTOME_ROLE_OF_LAT2_NTAL_LAB_ON_CALCIUM_MOBILIZATION | 0.979560111 | 5.8E-09 |
|  |  | REACTOME_FCGR_ACTIVATION | 0.973311856 | 5.8E-09 |
|  |  | REACTOME_SCAVENGING_OF_HEME_FROM_PLASMA | 0.96724413 | 5.8E-09 |
|  |  | REACTOME_ROLE_OF_PHOSPHOLIPIDS_IN_PHAGOCYTOSIS | 0.965142573 | 5.8E-09 |
|  |  | REACTOME_ANTIGEN_ACTIVATES_B_CELL_RECEPTOR_BCR_LEADING_TO_GENERATION_OF_SECOND_MESSENGERS | 0.962091714 | 5.8E-09 |
|  |  | REACTOME_CREATION_OF_C4_AND_C2_ACTIVATORS | 0.961372529 | 5.8E-09 |
|  |  | REACTOME_FCERI_MEDIATED_MAPK_ACTIVATION | 0.960932329 | 5.8E-09 |
|  |  | REACTOME_FCERI_MEDIATED_CA_2_MOBILIZATION | 0.952407422 | 5.8E-09 |
|  |  | REACTOME_FCGR3A_MEDIATED_IL10_SYNTHESIS | 0.951619346 | 5.8E-09 |
|  |  |  |  |  |
|  | KIRP | REACTOME_CD22_MEDIATED_BCR_REGULATION | 0.98577762 | 6.94E-09 |
|  |  | REACTOME_ROLE_OF_LAT2_NTAL_LAB_ON_CALCIUM_MOBILIZATION | 0.979320634 | 6.94E-09 |
|  |  | REACTOME_SCAVENGING_OF_HEME_FROM_PLASMA | 0.978955597 | 6.94E-09 |
|  |  | REACTOME_FCGR_ACTIVATION | 0.970167681 | 6.94E-09 |
|  |  | REACTOME_FCERI_MEDIATED_MAPK_ACTIVATION | 0.966858054 | 6.94E-09 |
|  |  | REACTOME_CREATION_OF_C4_AND_C2_ACTIVATORS | 0.965465144 | 6.94E-09 |
|  |  | REACTOME_ROLE_OF_PHOSPHOLIPIDS_IN_PHAGOCYTOSIS | 0.964801877 | 6.94E-09 |
|  |  | REACTOME_ANTIGEN_ACTIVATES_B_CELL_RECEPTOR_BCR_LEADING_TO_GENERATION_OF_SECOND_MESSENGERS | 0.95883393 | 6.94E-09 |
|  |  | REACTOME_FCERI_MEDIATED_CA_2_MOBILIZATION | 0.957252463 | 6.94E-09 |
|  |  |  |  |  |
|  | LUAD | REACTOME_FCGR_ACTIVATION | 0.938914431 | 8.57E-09 |
|  |  | REACTOME_CD22_MEDIATED_BCR_REGULATION | 0.936640598 | 8.57E-09 |
|  |  | REACTOME_CREATION_OF_C4_AND_C2_ACTIVATORS | 0.93368053 | 8.57E-09 |
|  |  | REACTOME_ROLE_OF_LAT2_NTAL_LAB_ON_CALCIUM_MOBILIZATION | 0.933438983 | 8.57E-09 |
|  |  | REACTOME_SCAVENGING_OF_HEME_FROM_PLASMA | 0.930276348 | 8.57E-09 |
|  |  | REACTOME_INITIAL_TRIGGERING_OF_COMPLEMENT | 0.925373851 | 8.57E-09 |
|  |  | REACTOME_ROLE_OF_PHOSPHOLIPIDS_IN_PHAGOCYTOSIS | 0.921879497 | 8.57E-09 |
|  |  | REACTOME_FCERI_MEDIATED_MAPK_ACTIVATION | 0.909576607 | 8.57E-09 |
|  |  | REACTOME_FCGR3A_MEDIATED_IL10_SYNTHESIS | 0.904237982 | 8.57E-09 |
|  |  | REACTOME_ANTIGEN_ACTIVATES_B_CELL_RECEPTOR_BCR_LEADING_TO_GENERATION_OF_SECOND_MESSENGERS | 0.901263663 | 8.57E-09 |
|  |  |  |  |  |
|  | OV | REACTOME_CD22_MEDIATED_BCR_REGULATION | 0.986868309 | 6E-09 |
|  |  | REACTOME_ROLE_OF_LAT2_NTAL_LAB_ON_CALCIUM_MOBILIZATION | 0.976092352 | 6E-09 |
|  |  | REACTOME_SCAVENGING_OF_HEME_FROM_PLASMA | 0.975266219 | 6E-09 |
|  |  | REACTOME_FCGR_ACTIVATION | 0.972581316 | 6E-09 |
|  |  | REACTOME_CREATION_OF_C4_AND_C2_ACTIVATORS | 0.969203462 | 6E-09 |
|  |  | REACTOME_ROLE_OF_PHOSPHOLIPIDS_IN_PHAGOCYTOSIS | 0.968503366 | 6E-09 |
|  |  | REACTOME_ANTIGEN_ACTIVATES_B_CELL_RECEPTOR_BCR_LEADING_TO_GENERATION_OF_SECOND_MESSENGERS | 0.967480513 | 6E-09 |
|  |  | REACTOME_FCERI_MEDIATED_MAPK_ACTIVATION | 0.964737617 | 6E-09 |
|  |  | REACTOME_FCERI_MEDIATED_CA_2_MOBILIZATION | 0.961184635 | 6E-09 |
|  |  | REACTOME_FCGR3A_MEDIATED_IL10_SYNTHESIS | 0.956751366 | 6E-09 |
|  |  |  |  |  |
|  | SARC | REACTOME_CD22_MEDIATED_BCR_REGULATION | 0.982628214 | 6.15E-09 |
|  |  | REACTOME_ROLE_OF_LAT2_NTAL_LAB_ON_CALCIUM_MOBILIZATION | 0.976229514 | 6.15E-09 |
|  |  | REACTOME_SCAVENGING_OF_HEME_FROM_PLASMA | 0.974797471 | 6.15E-09 |
|  |  | REACTOME_FCGR_ACTIVATION | 0.972305318 | 6.15E-09 |
|  |  | REACTOME_CREATION_OF_C4_AND_C2_ACTIVATORS | 0.972231245 | 6.15E-09 |
|  |  | REACTOME_ROLE_OF_PHOSPHOLIPIDS_IN_PHAGOCYTOSIS | 0.968874575 | 6.15E-09 |
|  |  | REACTOME_ANTIGEN_ACTIVATES_B_CELL_RECEPTOR_BCR_LEADING_TO_GENERATION_OF_SECOND_MESSENGERS | 0.957463952 | 6.15E-09 |
|  |  | REACTOME_FCERI_MEDIATED_MAPK_ACTIVATION | 0.956198548 | 6.15E-09 |
|  |  | REACTOME_FCGR3A_MEDIATED_IL10_SYNTHESIS | 0.955510416 | 6.15E-09 |
|  |  | REACTOME_INITIAL_TRIGGERING_OF_COMPLEMENT | 0.9545128 | 6.15E-09 |
|  |  |  |  |  |
|  | SKCM | REACTOME_CD22_MEDIATED_BCR_REGULATION | 0.97478524 | 6.15E-09 |
|  |  | REACTOME_SCAVENGING_OF_HEME_FROM_PLASMA | 0.972382814 | 6.15E-09 |
|  |  | REACTOME_ROLE_OF_LAT2_NTAL_LAB_ON_CALCIUM_MOBILIZATION | 0.970130342 | 6.15E-09 |
|  |  | REACTOME_PD_1_SIGNALING | 0.960432389 | 6.15E-09 |
|  |  | REACTOME_FCGR_ACTIVATION | 0.95889302 | 6.15E-09 |
|  |  | BIOCARTA_TCRA_PATHWAY | 0.957617912 | 3.05E-05 |
|  |  | REACTOME_CREATION_OF_C4_AND_C2_ACTIVATORS | 0.955233459 | 6.15E-09 |
|  |  | REACTOME_ROLE_OF_PHOSPHOLIPIDS_IN_PHAGOCYTOSIS | 0.954741846 | 6.15E-09 |
|  |  | REACTOME_ANTIGEN_ACTIVATES_B_CELL_RECEPTOR_BCR_LEADING_TO_GENERATION_OF_SECOND_MESSENGERS | 0.952741332 | 6.15E-09 |
|  |  | BIOCARTA_TCYTOTOXIC_PATHWAY | 0.947665877 | 0.000184 |
|  |  |  |  |  |
| FCRL6 | BRCA | REACTOME_CD22_MEDIATED_BCR_REGULATION | 0.969608824 | 3.15E-09 |
|  |  | REACTOME_PD_1_SIGNALING | 0.949346131 | 3.15E-09 |
|  |  | BIOCARTA_TCYTOTOXIC_PATHWAY | 0.946674303 | 7.01E-07 |
|  |  | BIOCARTA_THELPER_PATHWAY | 0.946579856 | 7.12E-07 |
|  |  | REACTOME_SCAVENGING_OF_HEME_FROM_PLASMA | 0.938794809 | 3.15E-09 |
|  |  | REACTOME_FCGR_ACTIVATION | 0.935613896 | 3.15E-09 |
|  |  | BIOCARTA_TCRA_PATHWAY | 0.933520167 | 4.8E-06 |
|  |  | BIOCARTA_BLYMPHOCYTE_PATHWAY | 0.933349643 | 4.88E-06 |
|  |  | REACTOME_CREATION_OF_C4_AND_C2_ACTIVATORS | 0.93328258 | 3.15E-09 |
|  |  | BIOCARTA_ASBCELL_PATHWAY | 0.932647801 | 2.92E-06 |
|  |  |  |  |  |
|  | CESC | BIOCARTA_TCRA_PATHWAY | 0.949056446 | 6.42E-08 |
|  |  | BIOCARTA_TCYTOTOXIC_PATHWAY | 0.948527636 | 7.55E-08 |
|  |  | BIOCARTA_THELPER_PATHWAY | 0.946700805 | 1E-07 |
|  |  | REACTOME_PD_1_SIGNALING | 0.943897499 | 3.71E-09 |
|  |  | REACTOME_CD22_MEDIATED_BCR_REGULATION | 0.942799337 | 3.71E-09 |
|  |  | BIOCARTA_CTLA4_PATHWAY | 0.932773243 | 3.71E-09 |
|  |  | REACTOME_FCGR_ACTIVATION | 0.925055257 | 3.71E-09 |
|  |  | BIOCARTA_ASBCELL_PATHWAY | 0.924505294 | 1.23E-06 |
|  |  | REACTOME_CREATION_OF_C4_AND_C2_ACTIVATORS | 0.922694832 | 3.71E-09 |
|  |  | BIOCARTA_BLYMPHOCYTE_PATHWAY | 0.921208416 | 3.11E-06 |
|  |  |  |  |  |
|  | HNSC | REACTOME_CD22_MEDIATED_BCR_REGULATION | 0.947496024 | 3.07E-09 |
|  |  | BIOCARTA_TCRA_PATHWAY | 0.939951749 | 5.53E-07 |
|  |  | REACTOME_PD_1_SIGNALING | 0.939574263 | 3.07E-09 |
|  |  | REACTOME_FCGR_ACTIVATION | 0.939393728 | 3.07E-09 |
|  |  | REACTOME_SCAVENGING_OF_HEME_FROM_PLASMA | 0.936458318 | 3.07E-09 |
|  |  | REACTOME_CREATION_OF_C4_AND_C2_ACTIVATORS | 0.934966386 | 3.07E-09 |
|  |  | REACTOME_INITIAL_TRIGGERING_OF_COMPLEMENT | 0.930356479 | 3.07E-09 |
|  |  | BIOCARTA_BLYMPHOCYTE_PATHWAY | 0.928926251 | 2.95E-06 |
|  |  | BIOCARTA_TCYTOTOXIC_PATHWAY | 0.928188898 | 3.22E-06 |
|  |  | BIOCARTA_THELPER_PATHWAY | 0.926359809 | 3.99E-06 |
|  |  |  |  |  |
|  | KIRP | REACTOME_CD22_MEDIATED_BCR_REGULATION | 0.977693502 | 2.8E-09 |
|  |  | REACTOME_ROLE_OF_LAT2_NTAL_LAB_ON_CALCIUM_MOBILIZATION | 0.96327213 | 2.8E-09 |
|  |  | REACTOME_FCGR_ACTIVATION | 0.961367808 | 2.8E-09 |
|  |  | REACTOME_SCAVENGING_OF_HEME_FROM_PLASMA | 0.960647751 | 2.8E-09 |
|  |  | BIOCARTA_TCRA_PATHWAY | 0.955020823 | 1.85E-08 |
|  |  | REACTOME_ROLE_OF_PHOSPHOLIPIDS_IN_PHAGOCYTOSIS | 0.953870091 | 2.8E-09 |
|  |  | REACTOME_FCERI_MEDIATED_MAPK_ACTIVATION | 0.945639524 | 2.8E-09 |
|  |  | BIOCARTA_CTLA4_PATHWAY | 0.945249058 | 2.8E-09 |
|  |  | BIOCARTA_TCYTOTOXIC_PATHWAY | 0.944846104 | 1.68E-07 |
|  |  | REACTOME_ANTIGEN_ACTIVATES_B_CELL_RECEPTOR_BCR_LEADING_TO_GENERATION_OF_SECOND_MESSENGERS | 0.944010256 | 2.8E-09 |
|  |  |  |  |  |
|  | LGG | BIOCARTA_TCYTOTOXIC_PATHWAY | 0.949236258 | 4.91E-07 |
|  |  | BIOCARTA_TCRA_PATHWAY | 0.939310883 | 2.94E-06 |
|  |  | BIOCARTA_THELPER_PATHWAY | 0.937258771 | 4.11E-06 |
|  |  | BIOCARTA_CTL_PATHWAY | 0.92690939 | 6.68E-06 |
|  |  | REACTOME_SCAVENGING_OF_HEME_FROM_PLASMA | 0.923845919 | 3.52E-09 |
|  |  | REACTOME_PD_1_SIGNALING | 0.92319627 | 3.52E-09 |
|  |  | REACTOME_CREATION_OF_C4_AND_C2_ACTIVATORS | 0.922359151 | 3.52E-09 |
|  |  | REACTOME_FCGR_ACTIVATION | 0.917538113 | 3.52E-09 |
|  |  | REACTOME_CD22_MEDIATED_BCR_REGULATION | 0.915947102 | 3.52E-09 |
|  |  | REACTOME_ROLE_OF_LAT2_NTAL_LAB_ON_CALCIUM_MOBILIZATION | 0.905915797 | 3.52E-09 |
|  |  |  |  |  |
|  | LUAD | BIOCARTA_TCYTOTOXIC_PATHWAY | 0.890800003 | 0.000905 |
|  |  | BIOCARTA_THELPER_PATHWAY | 0.890406695 | 0.000916 |
|  |  | BIOCARTA_BLYMPHOCYTE_PATHWAY | 0.867757578 | 0.002504 |
|  |  | BIOCARTA_TCRA_PATHWAY | 0.847599886 | 0.006571 |
|  |  | REACTOME_PD_1_SIGNALING | 0.844220327 | 6.68E-06 |
|  |  | BIOCARTA_CTLA4_PATHWAY | 0.839907292 | 0.000348 |
|  |  | REACTOME_RUNX1_AND_FOXP3_CONTROL_THE_DEVELOPMENT_OF_REGULATORY_T_LYMPHOCYTES_TREGS | 0.838970341 | 0.013109 |
|  |  | BIOCARTA_DC_PATHWAY | 0.831248146 | 0.001287 |
|  |  | REACTOME_CD22_MEDIATED_BCR_REGULATION | 0.823050575 | 6.09E-09 |
|  |  | REACTOME_GENERATION_OF_SECOND_MESSENGER_MOLECULES | 0.821449933 | 3.18E-07 |
|  |  |  |  |  |
|  | SKCM | REACTOME_PD_1_SIGNALING | 0.972075038 | 3.4E-09 |
|  |  | BIOCARTA_TCRA_PATHWAY | 0.965048525 | 6.9E-08 |
|  |  | REACTOME_CD22_MEDIATED_BCR_REGULATION | 0.95927539 | 3.4E-09 |
|  |  | REACTOME_FCGR_ACTIVATION | 0.948800452 | 3.4E-09 |
|  |  | REACTOME_SCAVENGING_OF_HEME_FROM_PLASMA | 0.947971694 | 3.4E-09 |
|  |  | BIOCARTA_TCYTOTOXIC_PATHWAY | 0.94400782 | 6.5E-06 |
|  |  | BIOCARTA_THELPER_PATHWAY | 0.942519686 | 7.74E-06 |
|  |  | REACTOME_ANTIGEN_ACTIVATES_B_CELL_RECEPTOR_BCR_LEADING_TO_GENERATION_OF_SECOND_MESSENGERS | 0.936980934 | 3.4E-09 |
|  |  | REACTOME_CREATION_OF_C4_AND_C2_ACTIVATORS | 0.9364761 | 3.4E-09 |
|  |  | REACTOME_ROLE_OF_PHOSPHOLIPIDS_IN_PHAGOCYTOSIS | 0.932266266 | 3.4E-09 |
|  |  |  |  |  |
|  | UCEC | REACTOME_CD22_MEDIATED_BCR_REGULATION | 0.967451906 | 3.41E-09 |
|  |  | BIOCARTA_TCYTOTOXIC_PATHWAY | 0.96000054 | 8.68E-08 |
|  |  | BIOCARTA_THELPER_PATHWAY | 0.959927911 | 9.02E-08 |
|  |  | REACTOME_FCGR_ACTIVATION | 0.954523584 | 3.41E-09 |
|  |  | BIOCARTA_TCRA_PATHWAY | 0.95437009 | 3.71E-07 |
|  |  | REACTOME_CREATION_OF_C4_AND_C2_ACTIVATORS | 0.949517409 | 3.41E-09 |
|  |  | REACTOME_PD_1_SIGNALING | 0.948369115 | 3.41E-09 |
|  |  | REACTOME_ROLE_OF_LAT2_NTAL_LAB_ON_CALCIUM_MOBILIZATION | 0.947931595 | 3.41E-09 |
|  |  | REACTOME_SCAVENGING_OF_HEME_FROM_PLASMA | 0.942725328 | 3.41E-09 |
|  |  | REACTOME_ROLE_OF_PHOSPHOLIPIDS_IN_PHAGOCYTOSIS | 0.939907533 | 3.41E-09 |
|  |  |  |  |  |
|  | UVM | BIOCARTA_TCRA_PATHWAY | 0.946781527 | 3.35E-08 |
|  |  | REACTOME_CD22_MEDIATED_BCR_REGULATION | 0.941844817 | 3.07E-09 |
|  |  | REACTOME_FCGR_ACTIVATION | 0.940533554 | 3.07E-09 |
|  |  | REACTOME_SCAVENGING_OF_HEME_FROM_PLASMA | 0.940113391 | 3.07E-09 |
|  |  | BIOCARTA_TCYTOTOXIC_PATHWAY | 0.931653788 | 3.23E-07 |
|  |  | REACTOME_ROLE_OF_PHOSPHOLIPIDS_IN_PHAGOCYTOSIS | 0.926694463 | 3.07E-09 |
|  |  | BIOCARTA_CTL_PATHWAY | 0.924411889 | 4.92E-07 |
|  |  | BIOCARTA_CTLA4_PATHWAY | 0.921375204 | 3.07E-09 |
|  |  | REACTOME_ROLE_OF_LAT2_NTAL_LAB_ON_CALCIUM_MOBILIZATION | 0.920129939 | 3.07E-09 |
|  |  | REACTOME_CREATION_OF_C4_AND_C2_ACTIVATORS | 0.918767962 | 3.07E-09 |
|  |  |  |  |  |
| FCRLA | BLCA | REACTOME_CD22_MEDIATED_BCR_REGULATION | 0.987627211 | 5.36E-09 |
|  |  | REACTOME_SCAVENGING_OF_HEME_FROM_PLASMA | 0.96222666 | 5.36E-09 |
|  |  | REACTOME_ROLE_OF_LAT2_NTAL_LAB_ON_CALCIUM_MOBILIZATION | 0.958520932 | 5.36E-09 |
|  |  | REACTOME_FCGR_ACTIVATION | 0.950895849 | 5.36E-09 |
|  |  | REACTOME_ANTIGEN_ACTIVATES_B_CELL_RECEPTOR_BCR_LEADING_TO_GENERATION_OF_SECOND_MESSENGERS | 0.949434382 | 5.36E-09 |
|  |  | REACTOME_CREATION_OF_C4_AND_C2_ACTIVATORS | 0.949095862 | 5.36E-09 |
|  |  | REACTOME_ROLE_OF_PHOSPHOLIPIDS_IN_PHAGOCYTOSIS | 0.944028017 | 5.36E-09 |
|  |  | REACTOME_FCERI_MEDIATED_MAPK_ACTIVATION | 0.939895955 | 5.36E-09 |
|  |  | REACTOME_FCERI_MEDIATED_CA_2_MOBILIZATION | 0.935536284 | 5.36E-09 |
|  |  | REACTOME_FCGR3A_MEDIATED_IL10_SYNTHESIS | 0.932676861 | 5.36E-09 |
|  |  |  |  |  |
|  | BRCA | REACTOME_CD22_MEDIATED_BCR_REGULATION | 0.982962532 | 4.27E-09 |
|  |  | REACTOME_ROLE_OF_LAT2_NTAL_LAB_ON_CALCIUM_MOBILIZATION | 0.963782675 | 4.27E-09 |
|  |  | REACTOME_FCGR_ACTIVATION | 0.962646086 | 4.27E-09 |
|  |  | REACTOME_SCAVENGING_OF_HEME_FROM_PLASMA | 0.962183802 | 4.27E-09 |
|  |  | REACTOME_CREATION_OF_C4_AND_C2_ACTIVATORS | 0.956617993 | 4.27E-09 |
|  |  | REACTOME_ROLE_OF_PHOSPHOLIPIDS_IN_PHAGOCYTOSIS | 0.956107106 | 4.27E-09 |
|  |  | REACTOME_PD_1_SIGNALING | 0.944263795 | 4.27E-09 |
|  |  | REACTOME_INITIAL_TRIGGERING_OF_COMPLEMENT | 0.94329329 | 4.27E-09 |
|  |  | REACTOME_ANTIGEN_ACTIVATES_B_CELL_RECEPTOR_BCR_LEADING_TO_GENERATION_OF_SECOND_MESSENGERS | 0.942521179 | 4.27E-09 |
|  |  | BIOCARTA_NO2IL12_PATHWAY | 0.937840798 | 5.73E-06 |
|  |  |  |  |  |
|  | HNSC | REACTOME_CD22_MEDIATED_BCR_REGULATION | 0.982962532 | 4.27E-09 |
|  |  | REACTOME_ROLE_OF_LAT2_NTAL_LAB_ON_CALCIUM_MOBILIZATION | 0.963782675 | 4.27E-09 |
|  |  | REACTOME_FCGR_ACTIVATION | 0.962646086 | 4.27E-09 |
|  |  | REACTOME_SCAVENGING_OF_HEME_FROM_PLASMA | 0.962183802 | 4.27E-09 |
|  |  | REACTOME_CREATION_OF_C4_AND_C2_ACTIVATORS | 0.956617993 | 4.27E-09 |
|  |  | REACTOME_ROLE_OF_PHOSPHOLIPIDS_IN_PHAGOCYTOSIS | 0.956107106 | 4.27E-09 |
|  |  | REACTOME_PD_1_SIGNALING | 0.944263795 | 4.27E-09 |
|  |  | REACTOME_INITIAL_TRIGGERING_OF_COMPLEMENT | 0.94329329 | 4.27E-09 |
|  |  | REACTOME_ANTIGEN_ACTIVATES_B_CELL_RECEPTOR_BCR_LEADING_TO_GENERATION_OF_SECOND_MESSENGERS | 0.942521179 | 4.27E-09 |
|  |  | BIOCARTA_NO2IL12_PATHWAY | 0.937840798 | 5.73E-06 |
|  |  |  |  |  |
|  | KICH | REACTOME_CD22_MEDIATED_BCR_REGULATION | 0.944434955 | 6.52E-09 |
|  |  | REACTOME_SCAVENGING_OF_HEME_FROM_PLASMA | 0.931083189 | 6.52E-09 |
|  |  | REACTOME_CREATION_OF_C4_AND_C2_ACTIVATORS | 0.926578382 | 6.52E-09 |
|  |  | REACTOME_FCGR_ACTIVATION | 0.926268925 | 6.52E-09 |
|  |  | REACTOME_ROLE_OF_LAT2_NTAL_LAB_ON_CALCIUM_MOBILIZATION | 0.922954654 | 6.52E-09 |
|  |  | REACTOME_ROLE_OF_PHOSPHOLIPIDS_IN_PHAGOCYTOSIS | 0.90721092 | 6.52E-09 |
|  |  | REACTOME_INITIAL_TRIGGERING_OF_COMPLEMENT | 0.90440776 | 6.52E-09 |
|  |  | REACTOME_FCERI_MEDIATED_MAPK_ACTIVATION | 0.894917558 | 6.52E-09 |
|  |  | REACTOME_ANTIGEN_ACTIVATES_B_CELL_RECEPTOR_BCR_LEADING_TO_GENERATION_OF_SECOND_MESSENGERS | 0.887660627 | 6.52E-09 |
|  |  | REACTOME_FCERI_MEDIATED_CA_2_MOBILIZATION | 0.886156485 | 6.52E-09 |
|  |  |  |  |  |
|  | LUAD | REACTOME_CD22_MEDIATED_BCR_REGULATION | 0.986736588 | 4.13E-09 |
|  |  | REACTOME_ROLE_OF_LAT2_NTAL_LAB_ON_CALCIUM_MOBILIZATION | 0.964757486 | 4.13E-09 |
|  |  | REACTOME_FCGR_ACTIVATION | 0.955129237 | 4.13E-09 |
|  |  | REACTOME_CREATION_OF_C4_AND_C2_ACTIVATORS | 0.95298712 | 4.13E-09 |
|  |  | REACTOME_SCAVENGING_OF_HEME_FROM_PLASMA | 0.946447303 | 4.13E-09 |
|  |  | REACTOME_ANTIGEN_ACTIVATES_B_CELL_RECEPTOR_BCR_LEADING_TO_GENERATION_OF_SECOND_MESSENGERS | 0.946334734 | 4.13E-09 |
|  |  | REACTOME_ROLE_OF_PHOSPHOLIPIDS_IN_PHAGOCYTOSIS | 0.94397196 | 4.13E-09 |
|  |  | REACTOME_INITIAL_TRIGGERING_OF_COMPLEMENT | 0.937215795 | 4.13E-09 |
|  |  | REACTOME_FCERI_MEDIATED_MAPK_ACTIVATION | 0.93370188 | 4.13E-09 |
|  |  | REACTOME_FCGR3A_MEDIATED_IL10_SYNTHESIS | 0.925480819 | 4.13E-09 |
|  |  |  |  |  |
|  | OV | REACTOME_CD22_MEDIATED_BCR_REGULATION | 0.982499574 | 5.36E-09 |
|  |  | REACTOME_SCAVENGING_OF_HEME_FROM_PLASMA | 0.970970892 | 5.36E-09 |
|  |  | REACTOME_FCGR_ACTIVATION | 0.970136885 | 5.36E-09 |
|  |  | REACTOME_ROLE_OF_LAT2_NTAL_LAB_ON_CALCIUM_MOBILIZATION | 0.964563822 | 5.36E-09 |
|  |  | REACTOME_ROLE_OF_PHOSPHOLIPIDS_IN_PHAGOCYTOSIS | 0.959936365 | 5.36E-09 |
|  |  | REACTOME_FCERI_MEDIATED_MAPK_ACTIVATION | 0.95923158 | 5.36E-09 |
|  |  | REACTOME_FCERI_MEDIATED_CA_2_MOBILIZATION | 0.958833794 | 5.36E-09 |
|  |  | REACTOME_CREATION_OF_C4_AND_C2_ACTIVATORS | 0.958217941 | 5.36E-09 |
|  |  | REACTOME_ANTIGEN_ACTIVATES_B_CELL_RECEPTOR_BCR_LEADING_TO_GENERATION_OF_SECOND_MESSENGERS | 0.955502117 | 5.36E-09 |
|  |  | REACTOME_INITIAL_TRIGGERING_OF_COMPLEMENT | 0.944327843 | 5.36E-09 |
|  |  |  |  |  |
|  | SARC | REACTOME_CD22_MEDIATED_BCR_REGULATION | 0.981592081 | 6.81E-09 |
|  |  | REACTOME_ROLE_OF_LAT2_NTAL_LAB_ON_CALCIUM_MOBILIZATION | 0.966136173 | 6.81E-09 |
|  |  | REACTOME_SCAVENGING_OF_HEME_FROM_PLASMA | 0.960009704 | 6.81E-09 |
|  |  | REACTOME_FCGR_ACTIVATION | 0.955003724 | 6.81E-09 |
|  |  | REACTOME_CREATION_OF_C4_AND_C2_ACTIVATORS | 0.953030221 | 6.81E-09 |
|  |  | REACTOME_ROLE_OF_PHOSPHOLIPIDS_IN_PHAGOCYTOSIS | 0.943002349 | 6.81E-09 |
|  |  | REACTOME_FCERI_MEDIATED_MAPK_ACTIVATION | 0.942578259 | 6.81E-09 |
|  |  | REACTOME_ANTIGEN_ACTIVATES_B_CELL_RECEPTOR_BCR_LEADING_TO_GENERATION_OF_SECOND_MESSENGERS | 0.940489325 | 6.81E-09 |
|  |  | BIOCARTA_TCRA_PATHWAY | 0.938428476 | 4.11E-05 |
|  |  | REACTOME_INITIAL_TRIGGERING_OF_COMPLEMENT | 0.934632395 | 6.81E-09 |
|  |  |  |  |  |
|  | UCEC | REACTOME_CD22_MEDIATED_BCR_REGULATION | 0.987795233 | 5.18E-09 |
|  |  | REACTOME_SCAVENGING_OF_HEME_FROM_PLASMA | 0.972639986 | 5.18E-09 |
|  |  | REACTOME_ROLE_OF_LAT2_NTAL_LAB_ON_CALCIUM_MOBILIZATION | 0.968085442 | 5.18E-09 |
|  |  | REACTOME_FCGR_ACTIVATION | 0.961499487 | 5.18E-09 |
|  |  | REACTOME_CREATION_OF_C4_AND_C2_ACTIVATORS | 0.954817835 | 5.18E-09 |
|  |  | REACTOME_ROLE_OF_PHOSPHOLIPIDS_IN_PHAGOCYTOSIS | 0.954234843 | 5.18E-09 |
|  |  | REACTOME_ANTIGEN_ACTIVATES_B_CELL_RECEPTOR_BCR_LEADING_TO_GENERATION_OF_SECOND_MESSENGERS | 0.95279456 | 5.18E-09 |
|  |  | REACTOME_FCERI_MEDIATED_MAPK_ACTIVATION | 0.951261065 | 5.18E-09 |
|  |  | REACTOME_FCERI_MEDIATED_CA_2_MOBILIZATION | 0.945916157 | 5.18E-09 |
|  |  | REACTOME_FCGR3A_MEDIATED_IL10_SYNTHESIS | 0.940352219 | 5.18E-09 |
|  |  |  |  |  |
| FCRLB | ACC | WP_CHOLESTEROL_BIOSYNTHESIS_PATHWAY | 0.888095505 | 3.43E-06 |
|  |  | WP_CHOLESTEROL_SYNTHESIS_DISORDERS | 0.872247226 | 3.34E-07 |
|  |  | REACTOME_ERYTHROCYTES_TAKE_UP_CARBON_DIOXIDE_AND_RELEASE_OXYGEN | 0.856358907 | 0.000145 |
|  |  | WP_MEVALONATE_ARM_OF_CHOLESTEROL_BIOSYNTHESIS_PATHWAY | 0.855540972 | 0.000149 |
|  |  | BIOCARTA_RANMS_PATHWAY | 0.829814828 | 0.004323 |
|  |  | KEGG_TERPENOID_BACKBONE_BIOSYNTHESIS | 0.819026912 | 0.000246 |
|  |  | REACTOME_CHOLESTEROL_BIOSYNTHESIS | 0.812373137 | 2.24E-07 |
|  |  | REACTOME_PRESYNAPTIC_NICOTINIC_ACETYLCHOLINE_RECEPTORS | 0.812089142 | 0.001965 |
|  |  | REACTOME_ACETYLCHOLINE_BINDING_AND_DOWNSTREAM_EVENTS | 0.800075976 | 0.001057 |
|  |  | REACTOME_HIGHLY_CALCIUM_PERMEABLE_POSTSYNAPTIC_NICOTINIC_ACETYLCHOLINE_RECEPTORS | 0.797359673 | 0.005159 |
|  |  |  |  |  |
|  | CESC | REACTOME_TYPE_I_HEMIDESMOSOME_ASSEMBLY | 0.894655351 | 0.000943 |
|  |  | REACTOME_CD22_MEDIATED_BCR_REGULATION | 0.877087263 | 9.34E-09 |
|  |  | REACTOME_FCGR_ACTIVATION | 0.86522733 | 9.34E-09 |
|  |  | BIOCARTA_CTL_PATHWAY | 0.8457865 | 0.001747 |
|  |  | BIOCARTA_THELPER_PATHWAY | 0.838959762 | 0.005076 |
|  |  | BIOCARTA_TCYTOTOXIC_PATHWAY | 0.838853376 | 0.005104 |
|  |  | REACTOME_CREATION_OF_C4_AND_C2_ACTIVATORS | 0.827242072 | 9.34E-09 |
|  |  | WP_TYPE_III_INTERFERON_SIGNALING | 0.826812732 | 0.020473 |
|  |  | REACTOME_ROLE_OF_LAT2_NTAL_LAB_ON_CALCIUM_MOBILIZATION | 0.818234931 | 9.34E-09 |
|  |  | REACTOME_ROLE_OF_PHOSPHOLIPIDS_IN_PHAGOCYTOSIS | 0.815220035 | 9.34E-09 |
|  |  |  |  |  |
|  | COAD | WP_LEPTIN_AND_ADIPONECTIN | 0.85308173 | 0.001867 |
|  |  | WP_MIR5093P_ALTERATION_OF_YAP1ECM_AXIS | 0.849329966 | 1.38E-05 |
|  |  | BIOCARTA_CLASSIC_PATHWAY | 0.809873873 | 0.002089 |
|  |  | BIOCARTA_GRANULOCYTES_PATHWAY | 0.794151517 | 0.002041 |
|  |  | REACTOME_CRMPS_IN_SEMA3A_SIGNALING | 0.793504933 | 0.000712 |
|  |  | REACTOME_CHYLOMICRON_REMODELING | 0.790972745 | 0.020023 |
|  |  | REACTOME_SCAVENGING_BY_CLASS_A_RECEPTORS | 0.787284433 | 0.000449 |
|  |  | BIOCARTA_NPP1_PATHWAY | 0.786072585 | 0.022817 |
|  |  | REACTOME_CROSSLINKING_OF_COLLAGEN_FIBRILS | 0.785629272 | 0.000972 |
|  |  | REACTOME_INTERACTION_BETWEEN_L1_AND_ANKYRINS | 0.779376217 | 3.71E-06 |
|  |  |  |  |  |
|  | GBM | REACTOME_IONOTROPIC_ACTIVITY_OF_KAINATE_RECEPTORS | 0.793896628 | 0.018606 |
|  |  | WP_AEROBIC_GLYCOLYSIS | 0.772803228 | 0.025057 |
|  |  | REACTOME_POLO_LIKE_KINASE_MEDIATED_EVENTS | 0.753182155 | 0.002955 |
|  |  | REACTOME_CONDENSATION_OF_PROMETAPHASE_CHROMOSOMES | 0.732059747 | 0.05561 |
|  |  | REACTOME_UNWINDING_OF_DNA | 0.731547958 | 0.053841 |
|  |  | REACTOME_CAMK_IV_MEDIATED_PHOSPHORYLATION_OF_CREB | 0.710563031 | 0.127976 |
|  |  | REACTOME_GABA_SYNTHESIS_RELEASE_REUPTAKE_AND_DEGRADATION | 0.70525333 | 0.004844 |
|  |  | BIOCARTA_RB_PATHWAY | 0.69662451 | 0.067755 |
|  |  | WP_GANGLIO_SPHINGOLIPID_METABOLISM | 0.693558896 | 0.069575 |
|  |  | REACTOME_SUPPRESSION_OF_PHAGOSOMAL_MATURATION | 0.688413061 | 0.076434 |
|  |  |  |  |  |
|  | LUAD | REACTOME_CD22_MEDIATED_BCR_REGULATION | 0.913961937 | 6.15E-09 |
|  |  | REACTOME_FCGR_ACTIVATION | 0.900626512 | 6.15E-09 |
|  |  | REACTOME_ROLE_OF_LAT2_NTAL_LAB_ON_CALCIUM_MOBILIZATION | 0.898413981 | 6.15E-09 |
|  |  | REACTOME_CREATION_OF_C4_AND_C2_ACTIVATORS | 0.892035563 | 6.15E-09 |
|  |  | BIOCARTA_TCYTOTOXIC_PATHWAY | 0.879540266 | 0.00108 |
|  |  | BIOCARTA_THELPER_PATHWAY | 0.879081722 | 0.00113 |
|  |  | REACTOME_ROLE_OF_PHOSPHOLIPIDS_IN_PHAGOCYTOSIS | 0.878679179 | 6.15E-09 |
|  |  | WP_PATHOGENESIS_OF_SARSCOV2_MEDIATED_BY_NSP9NSP10_COMPLEX | 0.871762505 | 6.36E-07 |
|  |  | REACTOME_INITIAL_TRIGGERING_OF_COMPLEMENT | 0.869209869 | 6.15E-09 |
|  |  | WP_MIR5093P_ALTERATION_OF_YAP1ECM_AXIS | 0.864918316 | 2.89E-05 |
|  |  |  |  |  |
|  | READ | WP_LEPTIN_AND_ADIPONECTIN | 0.93489471 | 6.22E-05 |
|  |  | WP_SEROTONIN_AND_ANXIETY | 0.855936765 | 0.000244 |
|  |  | REACTOME_DISEASES_ASSOCIATED_WITH_SURFACTANT_METABOLISM | 0.839776406 | 0.01566 |
|  |  | REACTOME_CROSSLINKING_OF_COLLAGEN_FIBRILS | 0.834124834 | 0.00019 |
|  |  | WP_PLATELETMEDIATED_INTERACTIONS_WITH_VASCULAR_AND_CIRCULATING_CELLS | 0.832577672 | 0.000243 |
|  |  | BIOCARTA_FIBRINOLYSIS_PATHWAY | 0.831690029 | 0.00346 |
|  |  | REACTOME_KERATAN_SULFATE_DEGRADATION | 0.829535353 | 0.003059 |
|  |  | PID_VEGF_VEGFR_PATHWAY | 0.828993726 | 0.020923 |
|  |  | REACTOME_STRIATED_MUSCLE_CONTRACTION | 0.825744657 | 6.9E-08 |
|  |  | BIOCARTA_GRANULOCYTES_PATHWAY | 0.820535232 | 0.002206 |
|  |  |  |  |  |
|  | STAD | BIOCARTA_EXTRINSIC_PATHWAY | 0.798242401 | 0.007151 |
|  |  | REACTOME_REMOVAL_OF_AMINOTERMINAL_PROPEPTIDES_FROM_GAMMA_CARBOXYLATED_PROTEINS | 0.787546461 | 0.03533 |
|  |  | WP_STEROID_BIOSYNTHESIS | 0.786523331 | 0.03717 |
|  |  | REACTOME_GLUCOCORTICOID_BIOSYNTHESIS | 0.784247583 | 0.039204 |
|  |  | REACTOME_CHYLOMICRON_ASSEMBLY | 0.776176124 | 0.049261 |
|  |  | REACTOME_COMMON_PATHWAY_OF_FIBRIN_CLOT_FORMATION | 0.773258396 | 0.000409 |
|  |  | REACTOME_GAMMA_CARBOXYLATION_TRANSPORT_AND_AMINO_TERMINAL_CLEAVAGE_OF_PROTEINS | 0.759530811 | 0.031744 |
|  |  | REACTOME_CHYLOMICRON_REMODELING | 0.757025137 | 0.085422 |
|  |  | REACTOME_UPTAKE_OF_DIETARY_COBALAMINS_INTO_ENTEROCYTES | 0.753492291 | 0.089893 |
|  |  | WP_VITAMIN_K_METABOLISM_AND_ACTIVATION_OF_DEPENDENT_PROTEINS | 0.751756055 | 0.066639 |
|  |  |  |  |  |
|  | THCA | REACTOME_CD22_MEDIATED_BCR_REGULATION | 0.950899851 | 6.15E-09 |
|  |  | REACTOME_RUNX1_AND_FOXP3_CONTROL_THE_DEVELOPMENT_OF_REGULATORY_T_LYMPHOCYTES_TREGS | 0.937159933 | 0.000345 |
|  |  | BIOCARTA_TCRA_PATHWAY | 0.935444106 | 6.88E-05 |
|  |  | REACTOME_FCGR_ACTIVATION | 0.934833659 | 6.15E-09 |
|  |  | REACTOME_ROLE_OF_LAT2_NTAL_LAB_ON_CALCIUM_MOBILIZATION | 0.929867663 | 6.15E-09 |
|  |  | REACTOME_ANTIGEN_ACTIVATES_B_CELL_RECEPTOR_BCR_LEADING_TO_GENERATION_OF_SECOND_MESSENGERS | 0.919010442 | 6.15E-09 |
|  |  | BIOCARTA_CTLA4_PATHWAY | 0.918971663 | 1.87E-07 |
|  |  | REACTOME_ROLE_OF_PHOSPHOLIPIDS_IN_PHAGOCYTOSIS | 0.917826026 | 6.15E-09 |
|  |  | REACTOME_SCAVENGING_OF_HEME_FROM_PLASMA | 0.914314939 | 6.15E-09 |
|  |  | REACTOME_CREATION_OF_C4_AND_C2_ACTIVATORS | 0.91285495 | 6.15E-09 |
|  |  |  |  |  |
|  | THYM | REACTOME_CD22_MEDIATED_BCR_REGULATION | 0.871524333 | 3.39E-09 |
|  |  | REACTOME_EICOSANOIDS | 0.864290462 | 0.000353 |
|  |  | REACTOME_FCGR_ACTIVATION | 0.844022687 | 3.39E-09 |
|  |  | REACTOME_ROLE_OF_LAT2_NTAL_LAB_ON_CALCIUM_MOBILIZATION | 0.842021499 | 3.39E-09 |
|  |  | REACTOME_FATTY_ACIDS | 0.840638032 | 0.000317 |
|  |  | REACTOME_FGFR3_LIGAND_BINDING_AND_ACTIVATION | 0.840506671 | 0.000467 |
|  |  | REACTOME_CREATION_OF_C4_AND_C2_ACTIVATORS | 0.831260839 | 3.39E-09 |
|  |  | WP_OSTEOBLAST_SIGNALING | 0.826186822 | 0.000625 |
|  |  | REACTOME_SCAVENGING_OF_HEME_FROM_PLASMA | 0.823875804 | 3.39E-09 |
|  |  | REACTOME_RUNX1_AND_FOXP3_CONTROL_THE_DEVELOPMENT_OF_REGULATORY_T_LYMPHOCYTES_TREGS | 0.809194735 | 0.01265 |
